# Aluminum–Boron Bond Formation by Boron Ester Oxidative Addition at an Alumanyl Anion

**DOI:** 10.1021/acs.inorgchem.3c02566

**Published:** 2023-09-06

**Authors:** Han-Ying Liu, Mary F. Mahon, Michael S. Hill

**Affiliations:** Department of Chemistry, University of Bath, Claverton Down, Bath BA2 7AY, U.K.

## Abstract

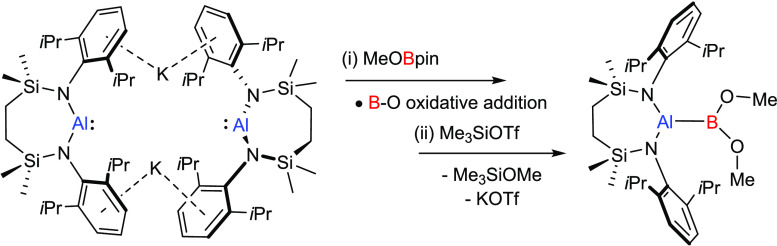

The potassium diamidoalumanyl, [K{Al(SiN^Dipp^)}]_2_ (SiN^Dipp^ = {CH_2_SiMe_2_NDipp}_2_), reacts with the terminal B–O bonds of pinacolato boron esters, ROBpin (R = Me, *i*-Pr), and B(OMe)_3_ to provide potsassium (alkoxy)borylaluminate derivatives, [K{Al(SiN^Dipp^)(OR)(Bpin)}]*_n_* (R = Me, *n* = 2; R = *i*-Pr, *n* = ∞) and [K{Al(SiN^Dipp^)(OMe)(B(OMe)_2_)}]_∞_, comprising Al–B σ bonds. An initial assay of the reactivity of these species with the heteroallene molecules, *N*,*N*′-diisopropylcarbodiimide and CO_2_, highlights the kinetic inaccessibility of their Al–B bonds; only decomposition at high temperature is observed with the carbodiimide, whereas CO_2_ preferentially inserts into the Al–O bond of [K{Al(SiN^Dipp^)(OMe)(Bpin)}]_2_ to provide a dimeric methyl carbonate species. Treatment of the acyclic dimethoxyboryl species, however, successfully liberates a terminal alumaboronic ester featuring trigonal N_2_Al–BO_2_ coordination environments at both boron and aluminum.

## Abstract 2

The potassium diamidoalumanyl, [K{Al(SiN^Dipp^)}]_2_ (SiN^Dipp^ = {CH_2_SiMe_2_NDipp}_2_), reacts with the terminal B−O bonds of pinacolato boron esters, ROBpin (R = OMe, O-*i*-Pr), and B(OMe)_3_ to provide potsassium (alkoxy)borylaluminate derivatives, [K{Al(SiN^Dipp^)(OR)(Bpin)}]*_n_* (R = OMe, *n* = 2; R = *i*-Pr, *n* = ∞) and [K{Al(SiN^Dipp^)(OMe)(B(OMe)_2_}]_∞_. While CO_2_ preferentially inserts into the Al−O bond of [K{Al(SiN^Dipp^)(OMe)(Bpin)}]_2_, treatment of the dimethoxyboryl species liberates a terminal alumaboronic ester, [(SiN^Dipp^)AlB(OMe)_2_], featuring trigonal coordination environments at both boron and aluminum.

## Introduction

**Figure 1:**
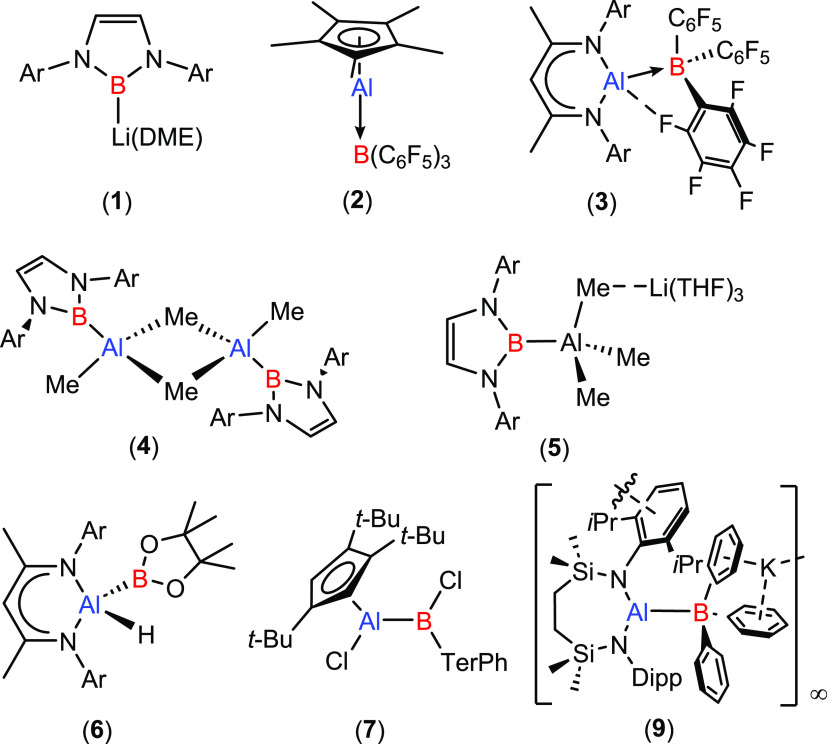
Yamashita and co-workers’ boryllithium (**1**) and exemplary compounds comprising unsupported Al–B bonding [Ar = 2,6-*i*-Pr_2_C_6_H_3_; TerPh = 2,6-(2,4,6-*i*-Pr_3_C_6_H_2_)_2_C_6_H_3_].

The identification of viable new borylation protocols is an important undertaking with relevance to a wide variety of onward cross-coupling processes and materials science applications.^1−10^ Although much organoborane synthesis remains dependent on the uncatalyzed or catalyzed hydroboration of C–E multiple bonds with electrophilic B–H-containing molecules,^11,12^ the last quarter century has witnessed significant progress in the development of borylation methods effected through the delivery of a formal boron nucleophile.^13−16^ While a historic majority of this chemistry has been achieved through the generation of an isolable or intermediate transition metal boryl,^17−21^ Yamashita and Nozaki’s realization of the sterically encumbered lithium boryl (**1**, Figure 1) in 2006 stimulated significant efforts to identify further M–B bonded molecules,^22−24^ where M represents one of the more electropositive elements of the periodic groups 1, 2, or 13.

More specifically, the generation of unsupported Al-to-B bonding has received significant attention. This was initially achieved by Cowley and co-workers through the synthesis of [(η^5^-C_5_Me_5_)Al–B(C_6_F_5_)_3_] (**2**),^25^ which is best considered as a Lewis acid adduct of the charge-neutral species, Cp*Al(I).^26^ Several closely related Cp*Al-to-boron adducts were subsequently described by Piers and co-workers,^27^ while Roesky prepared a similar compound (**3**) in which a β-diketiminate ligand was employed to support the formally low oxidation state aluminum center.^28,29^ In a complementary manner, Braunschweig and co-workers have developed an approach that invokes B → Al bond formation, which is achieved by coordination of the parent hydroborylene, (CAAC)(Me_3_P)BH (CAAC = cyclic alkyl(amino)carbene), to AlCl_3_.^30^ Covalent Al–B σ bond formation has been achieved either by exploiting the nucleophilic character of boryl anions such as **1** in the synthesis of a variety of boryl-substituted halo- and organoaluminum species (e.g., **4** and **5**),^31−38^ or, more recently, through the direct reductive coupling of an aluminum diiodide and a geometrically constrained triorganoborane.^39^ Of most relevance to the current study, however, are compounds **6** and **7**,^40,41^ in which Nikonov and Braunschweig, respectively, exploited the highly reducing nature of β-diketiminato and cyclopentadienyl Al(I) centers to effect B–H and B–X (X = halide) oxidative addition.^42^

**Scheme 1:**
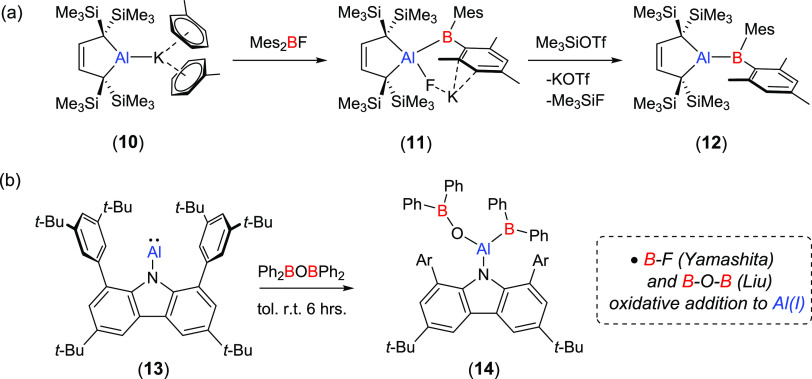
(a) Yamashita and Co-Workers’ Synthesis of the Tetraorganoalumaborane (**12**); (b) Zhang and Liu’s Synthesis of the Carbazolyl Alumaborane (**14**)

Our own interest centers on the reactivity of the cyclic potassium diamidoalumanyl, [K{Al(SiN^Dipp^)}]_2_ (**8**, SiN^Dipp^ = {CH_2_SiMe_2_NDipp}_2_),^43−46^ which, in common with other such species,^47−53^ behaves either as a potent source of the aluminum(I) nucleophile or as a two-electron reductant. We have previously reported that **8** reacts with BPh_3_ to provide an adduct (**9**),^44^ which may be considered as an alumanylborate analogue of the Al → B bonded species exemplified by compounds **2** and **3**. In a manner reminiscent of the formation of compound **6**, Al–E bond formation has been achieved by oxidative addition of a variety of both acidic and hydridic E–H bonds (E = N, O, Si, P, C) to alumanyl anions.^52,54,55^ Koshino and Kinjo have also shown that treatment of a cyclic (alkyl)(amino)alumanyl with BH_3_·SMe_2_ results in the generation of a heteroatomic AlB_2_ ring exhibiting a σ-aromatic structure.^52^ Extending this reactivity beyond E–H bonds, Yamashita and co-workers have recently demonstrated that B-Al bond formation may be effected by addition of a dialkylalumanyl variant (**10**) to Mes_2_BF (Scheme 1a).^56^ Although this process initially resulted in formal B–F oxidative addition and formation of a borylfluoroaluminate (**11**), the highly reactive tetraorganoalumaborane (**12**) could be liberated by treatment with Me_3_SiOTf. Even more recently, Zhang and Liu have reported that, although unreactive toward BPh_3_, treatment of the carbazolyl aluminylene (**13**) with Ph_2_BOBPh_2_ proceeds by B–OC oxidative addition at the Al(I) center to provide the (boryloxy)(boryl)alumane (**14**).^57,58^ In this contribution, we extend this mode of reactivity to B–O oxidative addition through the reaction of **8** with readily available cyclic and acyclic borate esters. We also describe an initial assay of the reactivity of the resultant (boryl)(alkoxy)aluminates with heteroallenes and the transformation of a dimethoxyboryl derivative to a terminal alumaboronic ester featuring trigonal N_2_Al-BO_2_ coordination environments at both boron and aluminum.

## Results and Discussion

Although an initial reaction between compound **8** and 2-methoxy-4,4,5,5-tetramethyl-1,3,2-dioxaborolane (MeOBpin) in *d*_6_-benzene induced only slow consumption of the alumanyl reagent at room temperature, heating for 4 h at 60 °C encouraged a decolorization of the initially yellow benzene solution and the generation of a single new species (**15**, eq 1). Compound **15** was characterized by the emergence of a sharp singlet at δ 3.35 ppm in the resultant ^1^H NMR spectrum, which was assigned as a new methoxide environment on the basis of its (3H) integration relative to the signals attributable to the SiN^Dipp^ ligand. The methyl groups of the *N*-Dipp *iso-*propyl substituents manifested as a series of four (6H) doublet signals between δ 1.46 and 1.33 ppm, while the SiN^Dipp^ silylmethyl resonances appeared as two singlets of a similar intensity diagnostic of a diastereotopic disposition of the Si*Me*_2_ units. Although these observations are consistent with a loss of the plane of symmetry associated with the chelated diamide ligand of **8**, no evidence for disruption of the local symmetry about the Bpin moiety, the methyl groups of which resonated as a sharp singlet (12H) at δ 0.76 ppm, could be observed on the NMR timescale at room temperature. The ^11^B NMR resonance attributed to **15** (δ 34.4 ppm) was also consistent with the maintenance of the 3-coordinate geometry of the MeOBpin starting material.^59^ The breadth of this signal, in conjunction with the above-noted equivalence of the Bpin methyl substituents, was, thus, consistent with the generation of a fluxional species comprising a terminal Al–Bpin bond and about which the boryl substituent is free to rotate.
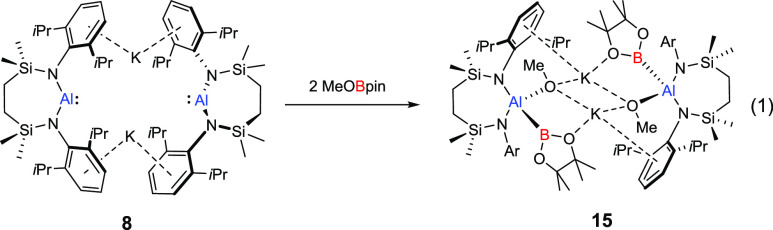
1

**Figure 2:**
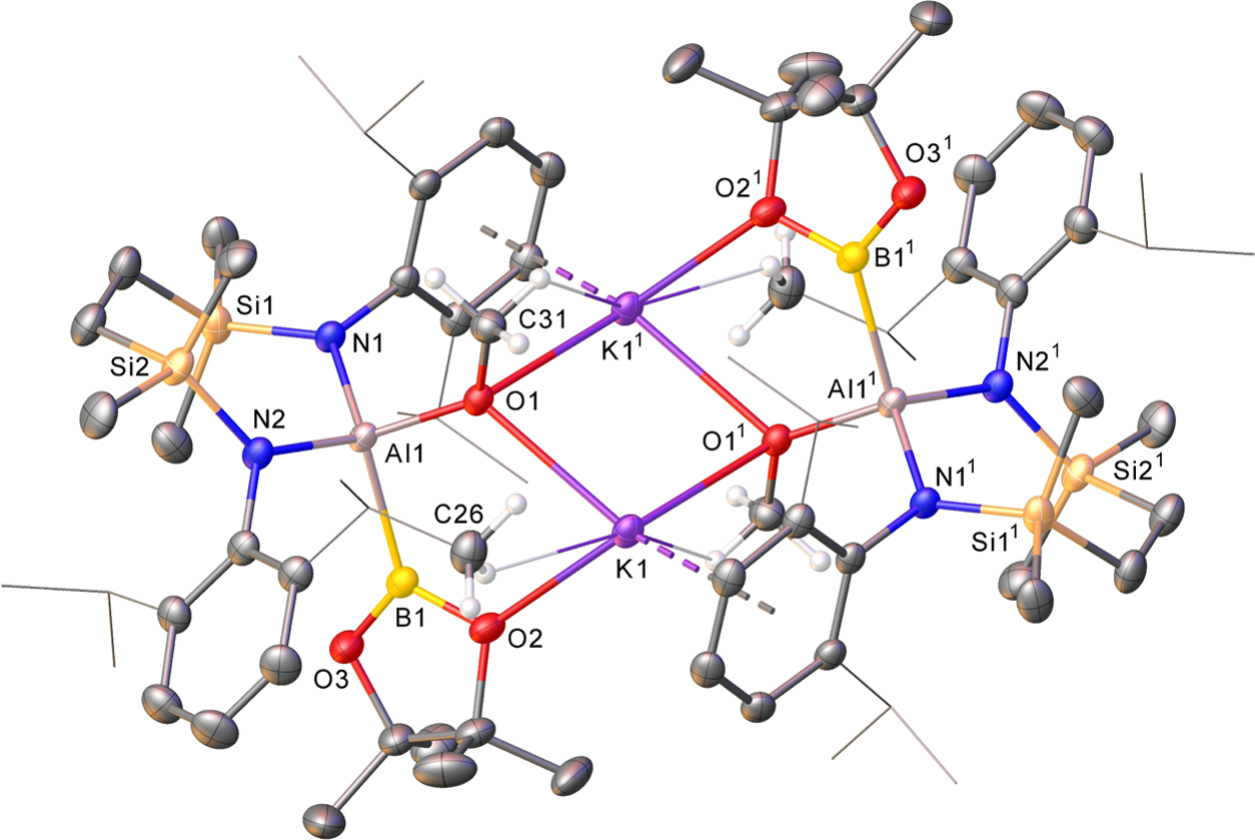
Displacement ellipsoid plot (30% probability) of compound **15**. For clarity, hydrogen atoms, apart from those relevant to close contacts with potassium, and a region of disordered solvent are omitted while the majority of *iso*-propyl substituents are shown as wireframes (also for visual ease). Symmetry operations to generate equivalent atoms: ^1^1 – *x*, 1 – *y*, 1 – *z*.

The veracity of these deductions was confirmed through the isolation of colorless single crystals of **15** isolated from a benzene/*n-*hexane solution at room temperature. The results of the X-ray diffraction analysis are depicted in Figure 2, while selected bond length and angle data are presented in Table 1. Compound **15** is a methoxy(boryl)aluminate in which the SiN^Dipp^ dianilide chelate is maintained with a charge balance upheld by K^+^ cations. The alkali metals propagate a centrosymmetric and dimeric structure such that each potassium is encapsulated by μ_2_-O-coordination through both methoxides, κ^1^-engagement with a single oxygen of each aluminum-bound Bpin unit and K-arene interactions with a single *N*-Dipp substituent of both SiN^Dipp^ ligands. The Al1–B1 bond [2.1716(18) Å] of **15** lies well within the range established for previously reported Al–B bonds [e.g., **2**, 2.169(3);^25^**3**, 2.183(5);^28^**4**, 2.119(3);^31^**6**, 2.1232(15);^40^**7**, 2.139(4);^41^**9**, 2.190(3);^44^**11**, 2.2805(19);^56^**12**, 2.191(2);^56^**14**, 2.143(4) Å^58^], irrespective of the 4-coordinate geometry at aluminum and its formal assignment as an aluminate center.

**Table 1:** Selected Bond Lengths (Å) and Angles (deg) of Compounds **15**–**17**, **19**, and **20**

	**15**	**16**	**17**	**19**	**20**
Al1–N1	1.8957(13)	1.9010(10)	1.8985(11)	1.8616(14)	1.797(6)^a^
Al1–N2	1.8878(13)	1.8920(10)	1.8932(11)	1.8714(14)	1.798(6)^b^
Al1–B1	2.1716(18)	2.1743(14)	2.1720(15)	2.169(2)	2.133(11)^c^
N1–Al1–N2	107.54(6)	105.68(4)	110.74(5)	112.26(6)	119.1(3)^d^
N1–Al1–B1	123.35(6)	122.25(5)	112.17(5)	106.62(7)	125.2(4)^e^
N2–Al1–B1	105.27(6)	105.07(5)	115.36(6)	122.56(7)	115.4(4)^f^

a Al2–N3 1.807(6).

b Al2–N4 1.792(6).

c Al2–B2 2.040(12).

d N3–Al2–N4 118.2(2).

e N3–Al2–B2 118.0(4).

f N4–Al2–B2 123.5(4).

**Figure 3:**
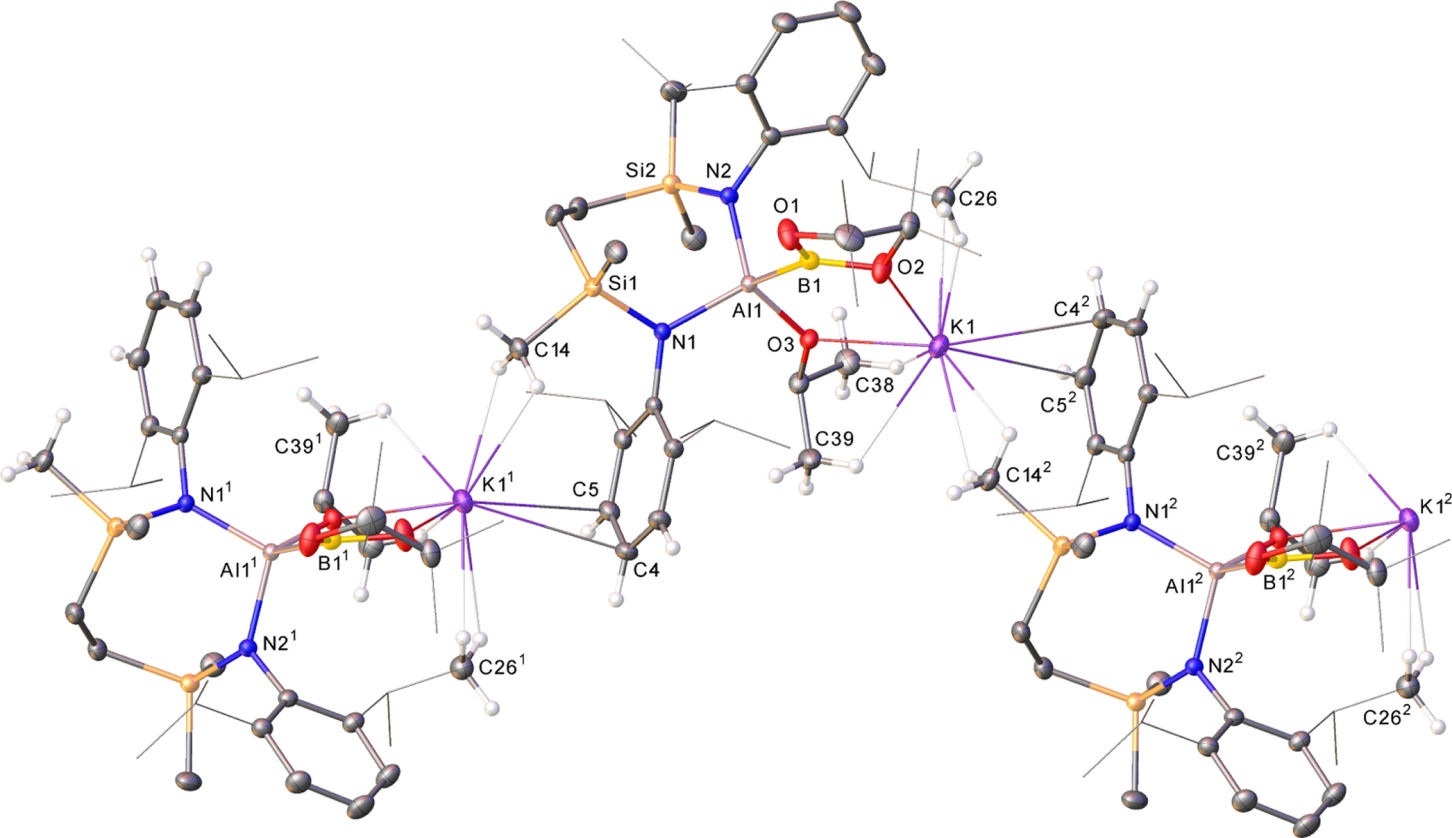
Displacement ellipsoid plot (30% probability) of a section of the 1-dimensional polymeric array propagated by compound **16**. Hydrogen atoms, apart from those relevant to close contacts with potassium, disordered atoms, and solvent are omitted for clarity, while Bpin methyl groups and *iso*-propyl substituents are shown as wireframes (also for visual ease). Symmetry operations to generate equivalent atoms: ^1^*x*, 3/2 – *y*, −1/2 + *z*; ^2^*x*, 3/2 – *y*, 1/2 + *z*; ^3^1 – *x*, 2 – *y*, 1 – *z*.

**Scheme 2:**
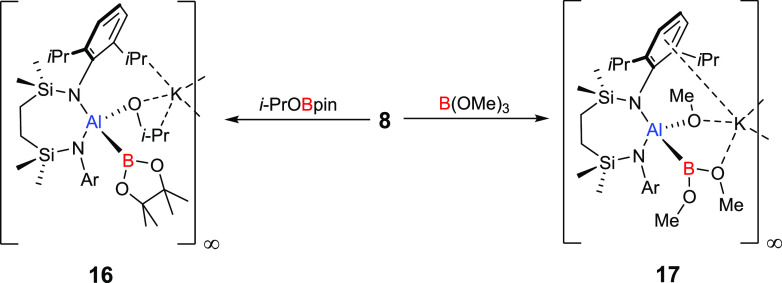
Synthesis of Compounds **16** and **17**

A further reaction performed between **8** and pinB(O*i*-Pr) in *d*_6_-benzene highlighted the kinetic impact of the more sterically encumbered boron ester, requiring 12 h at 60 °C to achieve the complete transformation of the alumanyl and the formation of compound **16** (Scheme 2). Although a ^11^B NMR resonance attributable to **16** could not be identified, the gross features of the corresponding ^1^H and ^13^C{^1^H} NMR spectra were reminiscent of those provided by compound **15** and were again consistent with a lowering of the local *C*_2_ symmetry about the aluminum center of **8**. This supposition was proved correct by a subsequent single-crystal X-ray diffraction analysis (Figure 3 and Table 1), albeit the resultant potassium *iso*-propoxy(boryl)aluminate describes a 1-dimensional polymeric array in the solid state. In this case, the two-fold μ_2_-O bridging intrinsic to the dimeric structure of **15** is evidently perturbed by the larger isopropoxide substituent such that each K^+^ ion now comprises a bidentate chelate structure with coordination provided by κ^1^-interactions with the isopropoxide and Bpin oxygen atoms of a single aluminate moiety. Consequently, the individual monomers interact *via* an infinite array of K-arene interactions and through close Si–CH_3_···K contacts with an adjacent SiN^Dipp^ aluminate structure. Despite this structural adjustment, the Al1–B1 bond of **16** [2.1743(14) Å] is effectively identical to that of **15**.

**Figure 4:**
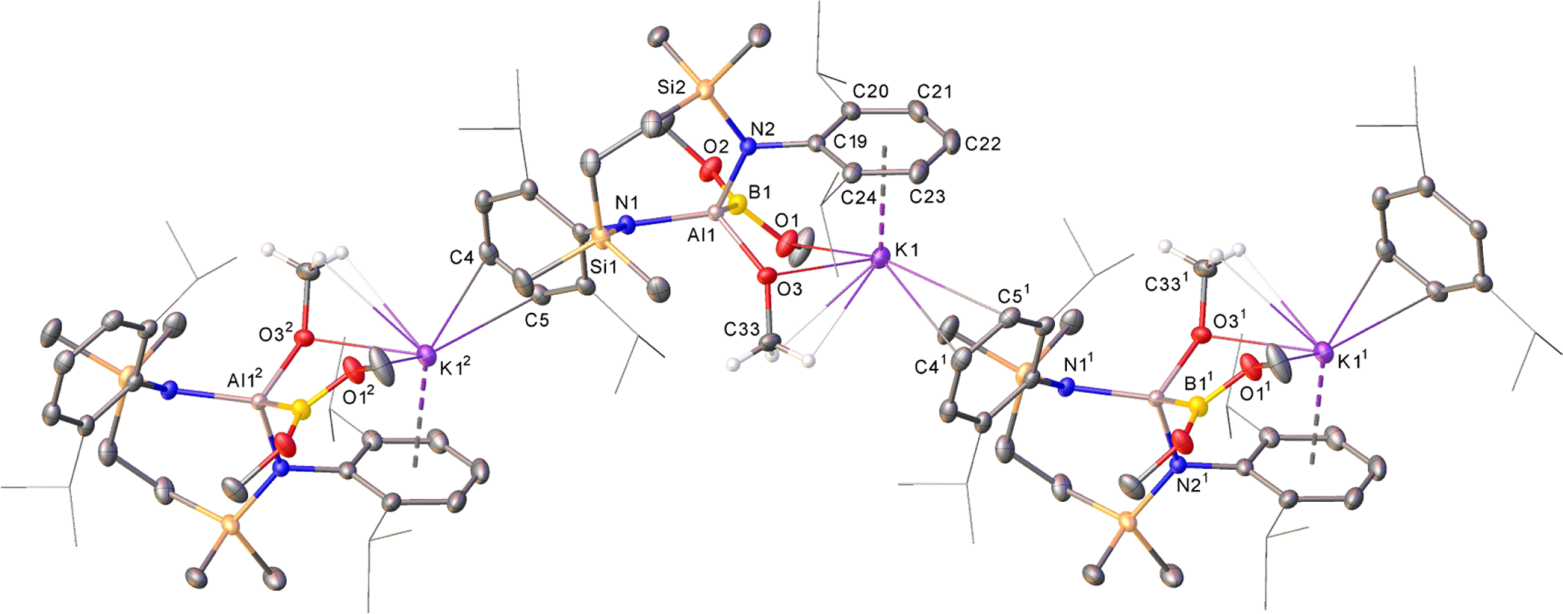
Displacement ellipsoid plot (30% probability) of a section of the 1-dimensional polymeric array propagated by compound **17**. For clarity, hydrogen atoms, apart from those relevant to close contacts with potassium, are omitted, and *iso*-propyl substituents are shown as wireframes. Symmetry operations to generate equivalent atoms: ^1^1 – *x*, 1/2 + *y*, 1/2 – *z*; ^2^1 – *x*, −1/2 + *y*, 1/2 – *z*.

As an extension of this reactivity to an acyclic borate ester, compound **8** was reacted with B(OMe)_3_. In this case, the potassium alumanyl was completely consumed inside 6 h at room temperature to provide compound **17** (Scheme 2). Although the main features of the ^1^H NMR spectrum of compound **17** in *d*_6_-benzene were again consistent with the formation of a borylaluminate species, it was clearly discriminated from those provided by compounds **15** and **16** through the appearance of two characteristic singlet resonances at δ 3.50 (6H) and 2.42 (3H) ppm, which were readily assignable as the methyl signals of the newly formed Al–B(OMe)_2_ and Al–OMe environments, respectively. Similarly, a severely broadened ^11^B NMR resonance could be discriminated at δ 40.7 ppm, consistent with the generation of a trigonal Al-bonded boron environment. The solid-state structure of **17** confirmed the formation of the desired potassium methoxy(dimethoxyboryl)aluminate complex (Figure 4 and Table 1). Although not isostructural to compound **16**, **17** crystallizes as a similar 1-dimensional array, which propagates through a sequence of *N*-arene···K bridging interactions between the individual potassium borylaluminate monomers. In this case, however, each potassium interacts with its aluminate contact ion partner not only through the aluminum- and boron-bound methoxides [O1–K1 2.7492(11), O3–K1 2.6645(10) Å] but also *via* an intramolecular polyhapto engagement with an *N*-Dipp ligand substituent.

**Figure 5:**
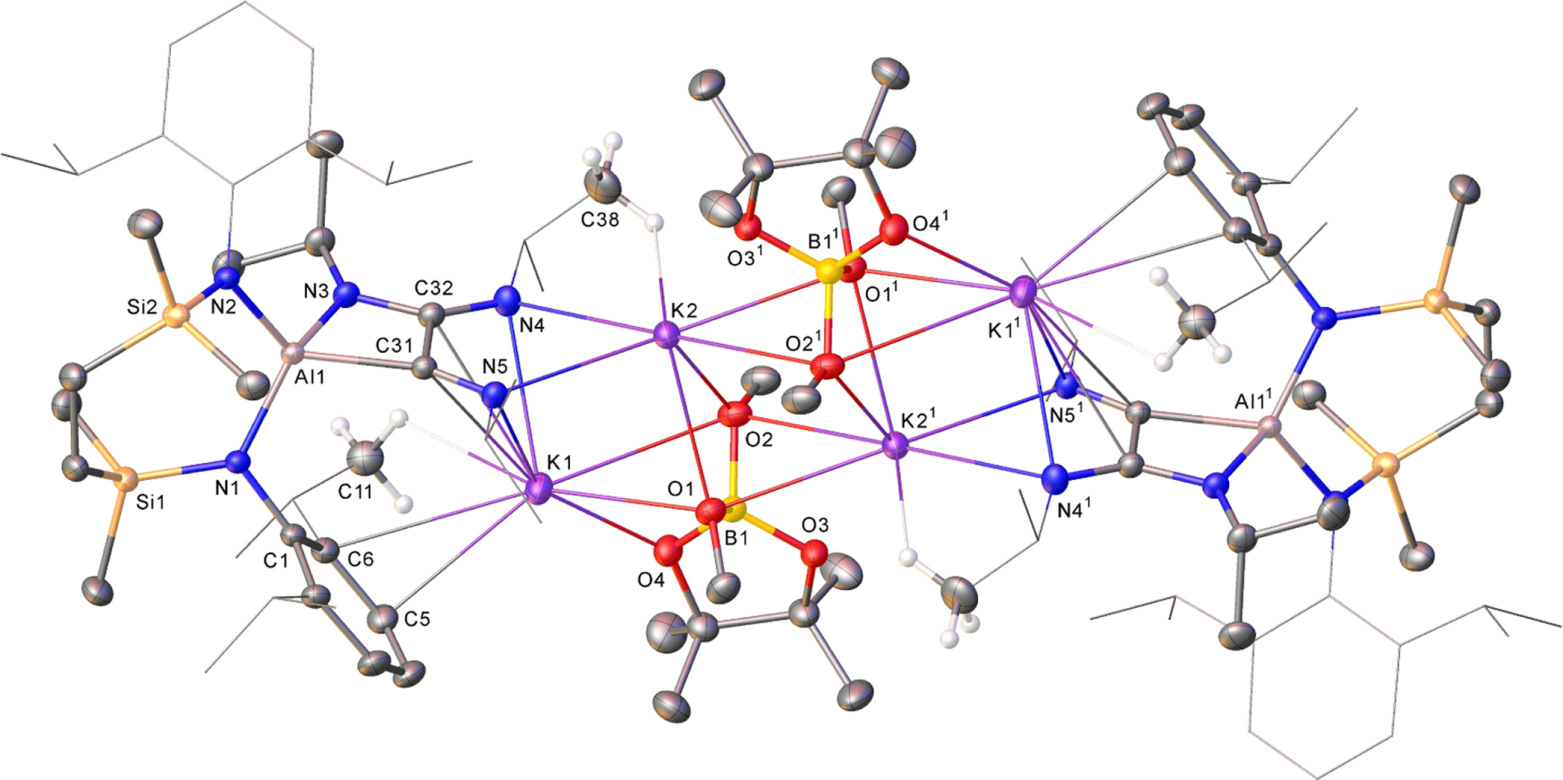
Displacement ellipsoid plot (30% probability) of compound **18**. Hydrogen atoms, apart from those relevant to close contacts with potassium, are omitted while selected Dipp and the majority of *iso*-propyl substituents are shown as wireframes for clarity. Symmetry operations to generate equivalent atoms: ^1^1 – *x*, 1 – *y*, −*z*.

We,^44,60,61^ and others,^62−64^ have previously employed the regiochemistry of carbodiimide insertion as a straightforward means with which to assess the relative polarization across an Al–M bond. Accordingly, the dimeric compound **15** was treated with 2 molar equiv of *N*,*N*′-diisopropylcarbodiimide in *d*_6_-benzene. Although no observable reaction could be discerned by ^1^H NMR spectroscopy after 1 week at 60 °C, further heating at 110 °C for 16 h induced the complete consumption of the borylaluminate. This more elevated temperature, however, resulted in the indiscriminate formation of an apparently complex mixture of products, whereupon a small number of colorless single crystals of compound **18** were deposited from the cooling reaction mixture. Although the resultant X-ray analysis allowed a tentative assignment of several diagnostic resonances in the ^1^H NMR spectrum arising from the initial mixture of products formed (see Supporting Information and Figure S17), compound **18** was identified to be a centrosymmetric potassium (imidoyl)amidinatoaluminate dimer, in which two monomeric [K{Al(SiN^Dipp^)(κ^2^-C,N-(*i*-PrN=C–C(N^i^Pr)_2_))}] units are effectively connected via 2 equiv of [K{(MeO)_2_Bpin}] (Figure 5).

**Scheme 3:**
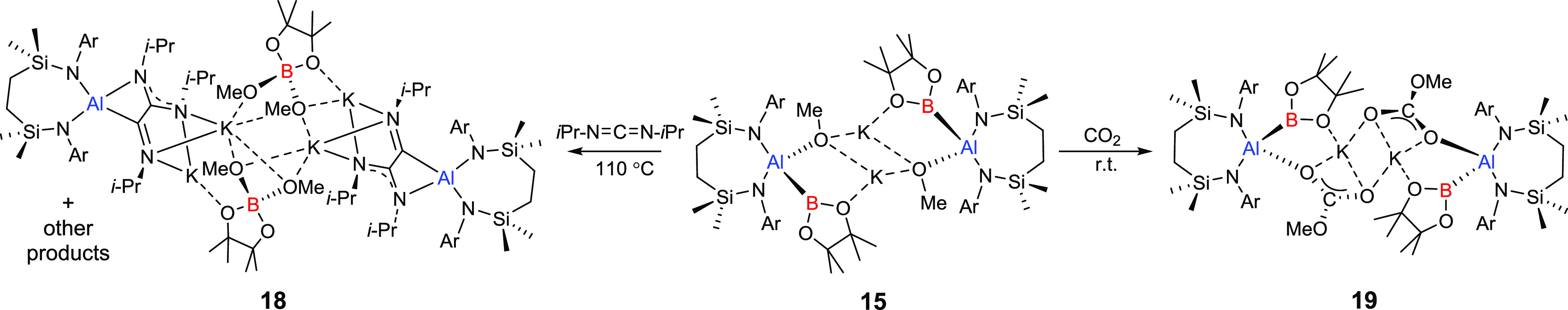
Synthesis of Compounds **18** and **19** (Ar = Dipp)

Although compound **18** could not be isolated in a sufficient bulk quantity to allow its complete characterization, and we cannot account for either the formal loss of an *i*-PrN unit or the C–C bond-forming process necessary for the formation of its (imidoyl)amidinate dianion, the structure of **18** is otherwise unremarkable. Its constitution, however, does indicate that any kinetic barrier toward insertion into the Al–B bond is too high to avoid the decomposition of both **15** and/or its carbodiimide reaction partner. On the assumption, therefore, that a more discriminating reaction could be induced by a less sterically encumbered heteroallene, compound **15** was then reacted with 2 atm of ^13^CO_2_ at room temperature in benzene. The reaction was complete under these conditions within 30 min, and compound **19** was most readily characterized in *d*_6_-benzene through the emergence of a doublet resonance (*J* = 4 Hz) at δ 3.43 ppm, integrating as 3 protons relative to the intensity of the various {SiN^Dipp^} silylmethyl and *iso*-propyl environments, in the resultant ^1^H NMR spectrum. These data and the correlation of this signal in an HMBC experiment with a ^13^C-labeled resonance at δ 156.4 ppm in the corresponding ^13^C NMR spectrum led us to identify **19** as a further borylaluminate derivative, but in which a molecule of CO_2_ had inserted into the Al–OMe bond of **15** to provide a methyl carbonate anion (Scheme 3).

**Figure 6:**
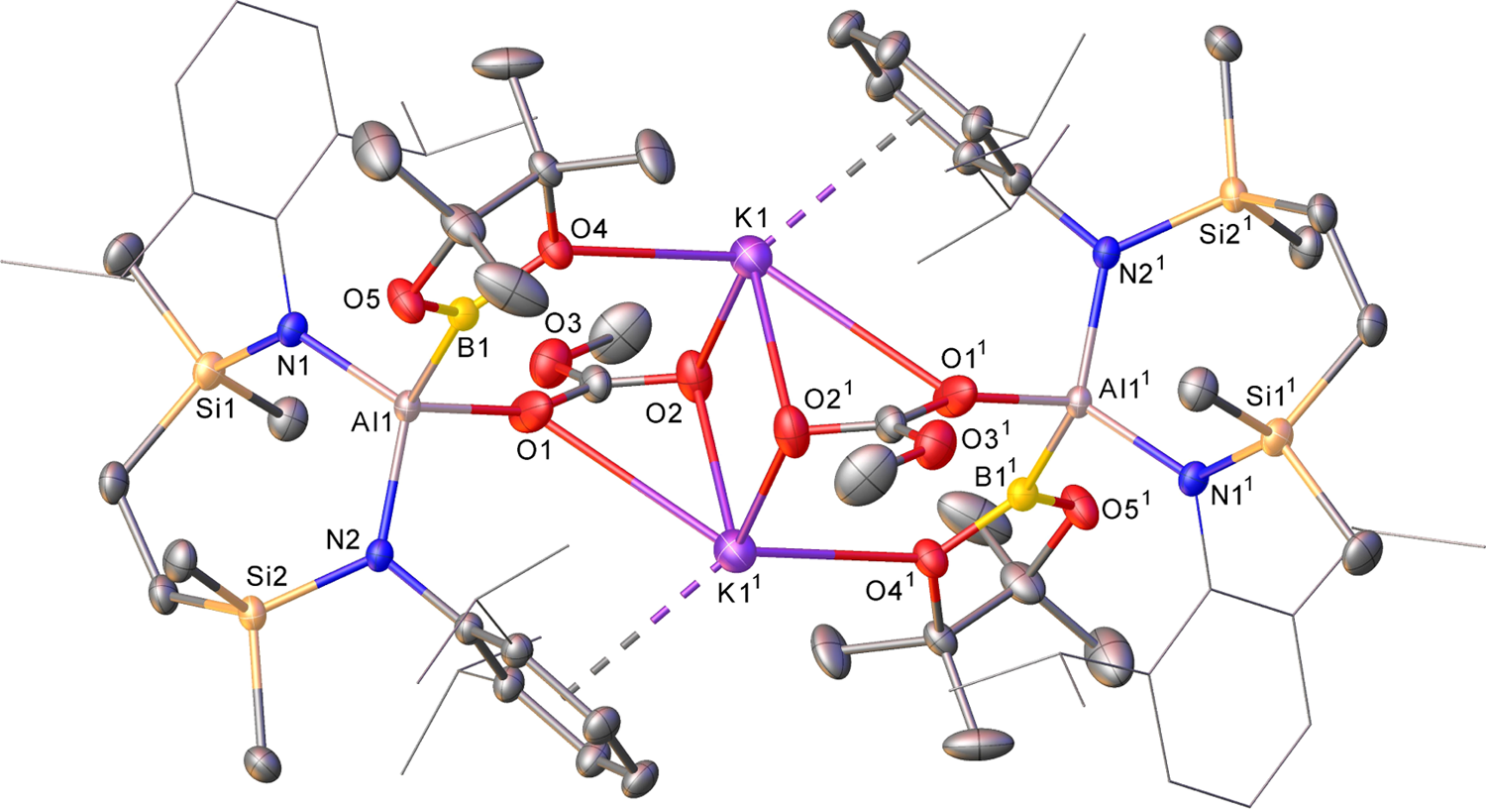
Displacement ellipsoid plot (30% probability) of compound **19**. For clarity, disordered atoms, occluded solvent, and hydrogen atoms are omitted. Selected Dipp and *iso*-propyl substituents are shown as wireframes, for visual ease. Symmetry operations to generate equivalent atoms: ^1^1 – *x*, 1 – *y*, 1 – *z*.

Colorless single crystals of **19** suitable for X-ray diffraction analysis were obtained by slow evaporation of a benzene/hexane solution at room temperature. The resultant structure, which confirmed the deductions of the solution-based analysis, is shown in Figure 6 with selected bond length and angle data provided in Table 1. The structure is a further centrosymmetric dimer in which two halves are connected by potassium centers that bridge to the aluminate component provided by both carbonate anions via κ^2^-O1,O2 and K1-μ_2_-O2-K1^1^ interactions. The dimer structure is consolidated by a close contact between each alkali metal ion and a single oxygen of each Bpin moiety [K1–O4 2.7590(14) Å]. The Al–B bond of **15** is maintained and is effectively unperturbed [Al1–B1 2.169(2) Å] by the incorporation of the AlO–CO_2_ unit.

**Figure 7:**
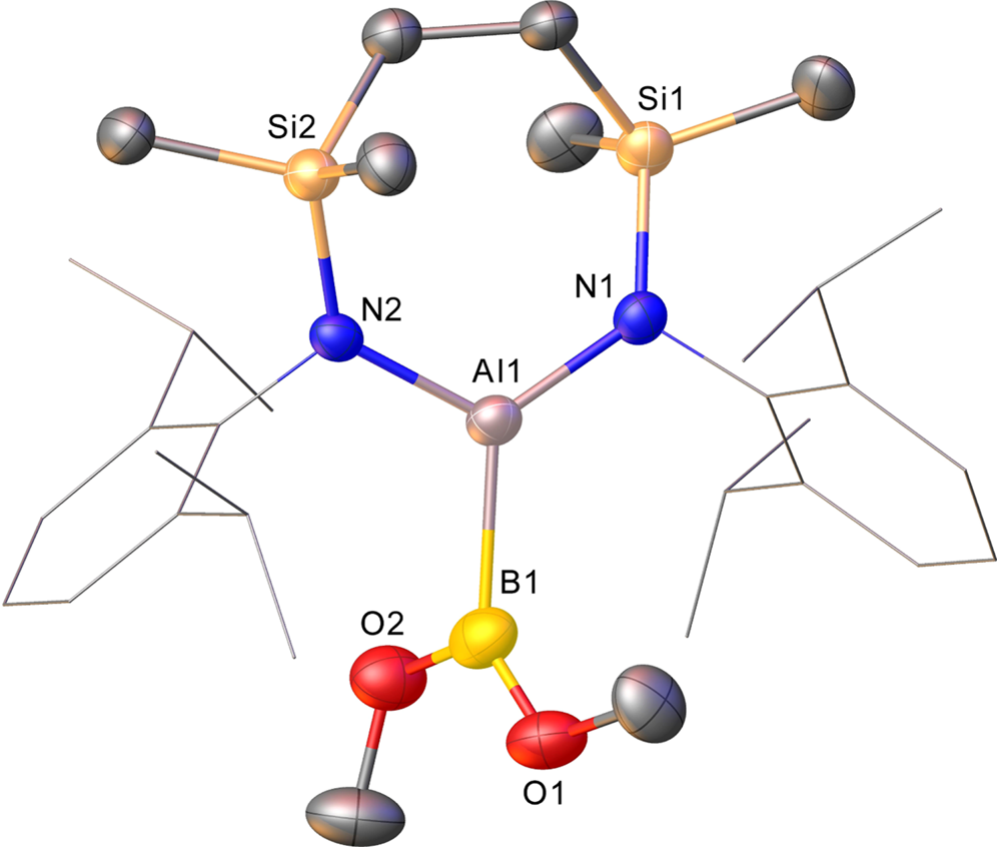
Displacement ellipsoid plot (25% probability) of the Al1-containing molecule in compound **20**. For clarity, disordered atoms, occluded solvent, and hydrogen atoms are omitted while Dipp substituents are shown as wireframes.

In the light of the formation of compound **19**, compound **15** was reacted with a single molar equivalent of Me_3_SiOTf in benzene. Although this treatment induced an immediate reaction, analysis of the resultant solution by ^1^H NMR spectroscopy highlighted the formation of a complex mixture of products. Somewhat surprisingly, a similar protocol applied to the acyclic borylaluminate **17** in *d*_6_-benzene provided significantly improved kinetic discrimination, the formation a colorless precipitate, assumed to be KOTf, and the generation of compound **20** (eq 2). The ^1^H and ^13^C NMR spectra of compound **20** were redolent of a C_2_ symmetric structure, while the observed ^11^B NMR chemical shift of δ 34.8 ppm confirmed the persistence of a 3-coordinate boron resultant environment. Filtration, removal of all volatiles, and crystallization of the colorless waxy solid from a mixed hexane/toluene solvent system provided single crystals of **20** suitable for X-ray diffraction analysis. Although the crystals were not of optimal quality, the consequent structure (Figure 7) confirmed the formation of a boryl aluminum species which, in a similar manner to Yamashita and co-workers’ recently reported derivative (**12**, Scheme 1a),^56^ presents both dissimilar group 13 centers with three-coordinate geometries. The asymmetric unit of compound **20** comprises two molecules, which, although constitutionally identical, display some notable variations across the individual bond metrics (Table 1). The Al–B bonds [Al1–B1 2.133(11), Al2–B2 2.040(12) Å] of both molecules are shorter than those of both the borylaluminate starting material **17** [2.1720(15) Å] and that reported for the most closely comparable derivative **12** [2.191(2) Å] in which both group 13 atoms are similarly three-coordinate. The elongation of this latter bond in **12** was ascribed to the high steric demands of both the aluminum- and boron-bound organic substituents, which also imposed a twisted relative disposition of the C–Al–C and C–B–C least squares planes [63.84°]. Despite the lower steric bulk of the methoxide substituents of compound **20**, the N–Al–N and O–B–O least square planes in both molecules subtend similar twist angles [62.1; 65.6°, for the Al1- and Al2-containing molecules, respectively], while examination of a space-filling model highlighted a high degree of steric protection afforded to the Al–B bond (see Figure S26). This latter feature was emphasized by the treatment of C_6_D_6_ solution of **20** with an atmosphere of ^13^CO_2_, which evidenced no consumption of the isotopically labeled reagent by ^13^C NMR spectroscopy even after extended periods at 110 °C, which only induced apparent decomposition of the boryl aluminum reagent.
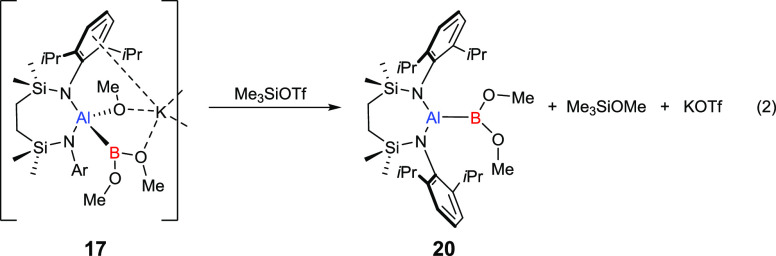
2

## Conclusions

The potassium diamidoalumanyl, [K{Al(SiN^Dipp^)}]_2_, reacts with the terminal B–O bonds of cyclic and acyclic boron esters to provide (alkoxy)borylaluminate derivatives comprising Al–B σ bonds. The solid-state nuclearity of the resultant compounds is dependent upon the identity and steric demands of the borate ester substituents, while an initial assay of the reactivity of these species with heteroallene molecules highlights the kinetic inaccessibility of their Al–B bonds. Treatment of the acyclic dimethoxyboryl species successfully liberates a molecular boryl aluminum diamide in which both group 13 centers present three-coordinate geometries. We are continuing to examine this chemistry and related reactivity.

## Experimental Section

### General Information

All experiments were conducted using standard Schlenk line and/or glovebox techniques under an inert atmosphere of argon. NMR spectra were recorded with a Bruker Avance III spectrometer (^1^H at 400 MHz, ^13^C at 101 MHz, ^11^B at 128 MHz). The spectra are referenced relative to residual protio solvent resonances. Elemental analyses were performed at Elemental Microanalysis Ltd., Okehampton, Devon, U.K. Solvents were dried by passage through a commercially available solvent purification system and stored under argon in ampoules over 4 Å molecular sieves. C_6_D_6_ was purchased from Sigma-Aldrich and dried over a potassium mirror before distilling and storage over molecular sieves. [{SiN^Dipp^}AlK]_2_ (**8**)^43^ was prepared according to the reported procedures. All other chemicals were purchased from Merck and used without further purification.

### Synthesis of Compound **15**

In a J Young’s tube, [K{Al(SiN^Dipp^)}]_2_ (**8**, 28 mg, 0.025 mmol) was dissolved in 0.4 mL of *d*_6_-benzene before the addition of 2-methoxy-4,4,5,5-tetramethyl-1,3,2-dioxaborolane (8.2 μL, 7.9 mg, 0.05 mmol) via a micropipette into the bright yellow solution. The resulting reaction mixture was kept at room temperature overnight (ca. 12 h) to afford a pale yellow solution, an *in situ*^1^H NMR spectrum indicates the reaction was not completed. The reaction mixture was, therefore, put at 60 °C for 4 h until the full conversion of the starting material. The colorless solution was then decanted into a sample vial and mixed with *n*-hexane (0.2 mL). Slow evaporation of the solution at room temperature then yielded colorless single crystals suitable for X-ray diffraction. The crystalline solids were then collected and washed with hexane (0.4 mL × 2) before all volatiles were removed *in vacuo*, providing compound **15** as a colorless powder. Yield 33 mg, 92%. Despite repeated attempts, an accurate microanalysis could not be obtained for this compound. ^1^H NMR (400 MHz, 298 K, Benzene-*d*_6_) δ 7.08 (dd, *J* = 7.5, 1.9 Hz, 2H, *m*-C_6_*H*_3_), 7.05 (dd, *J* = 7.5, 1.9 Hz, 2H, *m*-C_6_*H*_3_), 6.92 (t, *J* = 7.5 Hz, 2H, *p*-C_6_*H*_3_), 4.27 (sept, *J* = 6.8 Hz, 4H, C*H*Me_2_), 3.25 (s, 3H, OC*H*_3_), 1.46 (d, *J* = 6.8 Hz, 6H, CH*Me*_2_), 1.42* (d, *J* = 6.8 Hz, 6H, CH*Me*_2_), 1.41* (d, *J* = 6.8 Hz, 6H, CH*Me*_2_), 1.34 (s, 4H, SiC*H*_2_), 1.33 (d, *J* = 6.8 Hz, 6H, CH*Me*_2_), 0.76 (s, 12H, BOC*Me*_2_), 0.43 (s, 6H, Si*Me*_2_), 0.37 (s, 6H, Si*Me*_2_).*overlapping peaks. ^13^C{^1^H} NMR (101 MHz, 298 K, Benzene-*d*_6_) δ 151.1 (*i*-*C*_6_H_3_), 148.8 (*o*-*C*_6_H_3_), 148.5 (*o*-*C*_6_H_3_), 123.5 (*m*-*C*_6_H_3_), 123.3 (*m*-*C*_6_H_3_), 121.6 (*p*-*C*_6_H_3_), 80.3 (BO*C*Me_2_), 50.5 (O*C*H_3_), 27.5 (*C*HMe_2_), 27.4 (*C*HMe_2_), 26.6 (CH*Me*_2_), 26.4 (CH*Me*_2_), 26.4 (BOC*Me*_2_), 26.1 (CH*Me*_2_), 25.8 (CH*Me*_2_), 15.2 (Si*C*H_2_), 14.4 (Si*C*H_2_), 2.7 (Si*Me*_2_), 2.3 (Si*Me*_2_). ^11^B NMR (128 MHz, 298 K, Benzene-*d*_6_) δ 34.4 (very broad).

### Synthesis of Compound **16**

In a J Young’s tube, [K{Al(SiN^Dipp^)}]_2_ (**8**, 28 mg, 0.025 mmol) was dissolved in 0.4 mL of *d*_6_-benzene before the addition of 2-isopropoxy-4,4,5,5-tetramethyl-1,3,2-dioxaborolane (10.2 μL, 9.3 mg, 0.05 mmol) via a micropipette into the bright yellow solution. The resulting reaction mixture was then kept at 60 °C for 12 h, affording a colorless solution. The reaction mixture was then decanted into a sample vial and mixed with *n*-hexane (0.2 mL). Slow evaporation of the solution at room temperature then yielded colorless single crystals suitable for X-ray diffraction. The crystalline solids were then collected and washed with hexane (0.4 mL × 2) before all volatiles were removed *in vacuo*, providing compound 16 as a white powder. Yield 30 mg, 80%. Anal Calc’d for C_42_H_72_AlBN_2_O_3_Si_2_ (**16.** C_6_H_6_, 786.08): C, 64.17; H, 9.23; N, 3.56%. Found: C, 63.83; H, 8.97; N, 3.41%. ^1^H NMR (400 MHz, 298 K, Benzene-*d*_6_) δ 7.22–7.18 (m, 2H, C_6_*H*_3_), 7.14–7.05 (m, 4H, C_6_*H*_3_), 4.47 (sept, *J* = 6.8 Hz, 2H, C*H*Me_2_ on SiN^Dipp^), 4.33 (p, *J* = 6.8 Hz, 2H, C*H*Me_2_ on SiN^Dipp^), 4.13 (sept, *J* = 6.0 Hz, 1H, C*H*Me_2_ on O*i*Pr), 1.49 (d, *J* = 6.8 Hz, 6H, CH*Me*_2_ on SiN^Dipp^), 1.47 (d, *J* = 6.8 Hz, 6H, CH*Me*_2_ on SiN^Dipp^), 1.45 (s, 4H, SiC*H*_2_), 1.37 (d, *J* = 6.0 Hz, 6H, CH*Me*_2_ on O*i*Pr), 1.24 (d, *J* = 6.8 Hz, 12H, CH*Me*_2_ on SiN^Dipp^, *overlapping with hexane impurity), 0.76 (s, 12H, BOC*Me*_2_), 0.51 (s, 6H, Si*Me*_2_), 0.37 (s, 6H, Si*Me*_2_). ^13^C NMR (101 MHz, 298 K, Benzene-*d*_6_) δ 151.5 (*i*-*C*_6_H_3_), 147.7 (*o*-*C*_6_H_3_), 147.0 (*o*-*C*_6_H_3_), 124.0 (*m*-*C*_6_H_3_), 123.8 (*m*-*C*_6_H_3_), 121.6 (*p*-*C*_6_H_3_), 80.50 (BO*C*Me_2_), 61.8 (KO*C*HMe_2_), 27.3 (*C*HMe_2_), 27.3 (*C*HMe_2_), 26.9 (O CH*Me*_2_), 26.8 (CH*Me*_2_), 26.4 (CH*Me*_2_), 26.3 (CH*Me*_2_), 26.2 (CH*Me*_2_), 26.0 (BOC*Me*), 15.4 (Si*C*H_2_), 4.3 (Si*Me*_2_), 4.1 (Si*Me*_2_). ^11^B resonance correlated to Al–Bpin was not observed even at 1000 scans.

### Synthesis of Compound **17**

In a J Young’s tube, [K{Al(SiN^Dipp^)}]_2_ (**8**, 28 mg, 0.025 mmol) was dissolved in 0.4 mL of *d*_6_-benzene before the addition of trimethylborate (5.6 μL, 5.2 mg, 0.05 mmol) via a micropipette into the bright yellow solution. The resulting pale yellow reaction mixture was kept at room temperature for 6 h to afford a colorless solution, which was indicated by an *in situ*^1^H NMR spectrum to have quantitatively transformed into one singular species. The reaction mixture was then decanted into a sample vial in glovebox and layered with hexane (0.2 mL) at room temperature, giving single crystals suitable for X-ray diffraction. The crystalline solids were then collected and washed with hexane (0.4 mL × 2) before all volatiles were removed *in vacuo*, yielding compound **17** as a colorless powder. Yield 28 mg, 84%. Anal Calc’d for C_33_H_59_AlBN_2_O_3_Si_2_ (**17**, 664.91): C, 59.61; H, 8.94; N, 4.21%. Found: C, 60.18; H, 8.71; N, 4.02%. The NMR sample was then prepared by re-dissolving the obtained white powder in deuterated benzene. ^1^H NMR (400 MHz, 298 K, Benzene-*d*_6_) δ 7.13–7.00 (m, 4H, *m*-C_6_*H*_3_), 6.93–6.86 (m, 2H, *p*-C_6_*H*_3_), 4.29 (sept, *J* = 6.7 Hz, 2H, C*H*Me_2_), 4.10 (sept, *J* = 6.7 Hz, 2H, C*H*Me_2_), 3.50 (s, 6H, BO*Me*_2_), 2.42 (s, 3H, KO*Me*), 1.46 (d, *J* = 6.7 Hz, 6H, CH*Me*_2_), 1.40 (d, *J* = 6.7 Hz, 6H, CH*Me*_2_), 1.31–1.25 (m, 10H, CH*Me*_2_ and SiC*H*_2_, overlapping peaks), 1.23 (d, *J* = 6.7 Hz, 6H, CH*Me*_2_), 0.56 (s, 6H, Si*Me*_2_), 0.23 (s, 6H, Si*Me*_2_). ^13^C NMR (101 MHz, 298 K, Benzene-*d*_6_) δ 149.1 (*o*-*C*_6_H_3_), 148.1 (*i*-*C*_6_H_3_), 123.9 (*m*-*C*_6_H_3_), 123.8 (*m*-*C*_6_H_3_), 121.8 (*p*-*C*_6_H_3_), 51.4 (BO*Me*_2_, observed in HSQC), 49.3 (KO*Me*), 27.7 (*C*HMe_2_), 27.4 (*C*HMe_2_), 26.1 (CH*Me*_2_), 25.8 (CH*Me*_2_), 25.7 (CH*Me*_2_), 25.37 (CH*Me*_2_), 14.4 (Si*C*H_2_), 3.9 (Si*Me*_2_), 3.6 (Si*Me*_2_). ^11^B NMR (128 MHz, 298 K, Benzene-*d*_6_) δ 40.7.

### Isolation of [K{Al(SiN^Dipp^)(κ^2^-C,N-(*i*-PrN=C–C(N*^i^*Pr)_2_))}] (**18**); Reaction of [C{N*i*-Pr}_2_] with **8**

In a J Young’s tube, [K{Al(SiN^Dipp^)(OMe)(Bpin)}] (**8**, 34 mg, 0.05 mmol) was dissolved in 0.4 mL of *d*_6_-benzene before the addition of 2 equiv of *N,N*′-diisopropylcarbodiimide (15.6 μL, 12.6 mg, 0.1 mmol) *via* a micropipette into the colorless solution. No reaction was observed by ^1^H NMR spectroscopy when the reaction mixture was kept at 60 °C for a week. However, subtle but noticeable changes were observed in ^1^H NMR spectra of the reaction mixture when it was kept at 110 °C for 1 h. The reaction mixture was then maintained at 110 °C for additional 15 h, whereupon the standing mixture transformed into a cloudy colorless mixture with a tiny amount of colorless crystals, which were identified by X-ray diffraction analysis to be **18**. Additional 0.2 mL of *d*_6_-benezne dissolved all the crystals to provide a hazy colorless solution, allowing some diagnostic peaks of compound **18** to be identified by NMR spectroscopy. ^1^H NMR (400 MHz, 298 K, Benzene-*d*_6_) δ 7.01–6.87 (m, 6H, Ar*H*), 3.99 (sept, *J* = 6.8 Hz, 4H, C*H*Me_2_ of SiN^Dipp^), 3.29 (s, 3H, O*Me*), 2.84 (s, 3H, O*Me*), 2.51 (sept, *J* = 6.6 Hz, 3H, C*H*Me_2_ of C_2_{N^i^Pr}_3_), 0.98 (s, 12H, Bpin), 0.87 (s, 4H, SiC*H*_2_), 0.65 (d, *J* = 6.6 Hz, 9H, CH*Me*_2_ of C_2_{N^i^Pr}_3_), 0.27 (s, 12H, Si*Me*_2_); CH*Me*_2_ on SiN^Dipp^ was not identified due to complexity in δ_H_ 1.50–1.00. Multiple attempts to isolate compound **18** by recrystallization only provided a mixture of species.

### Synthesis of Compound **19**

In a J Young’s tube, [K{Al(SiN^Dipp^)(OMe)(Bpin)}] (**15**, 34 mg, 0.05 mmol) was dissolved in 0.4 mL of *d*_6_-benzene. The colorless solution was then degassed by three freeze–pump–thaw cycles before charged with 2 atm of ^13^CO_2_. Full conversion was observed by the ^1^H NMR spectroscopy within 30 min of the reaction. Colorless single crystals suitable for X-ray crystallography were obtained from a slow evaporation of a benzene/hexane solution at room temperature. Yield 28 mg, 66%. Despite repeated attempts, an accurate microanalysis could not be obtained for this compound. ^1^H NMR (400 MHz, 298 K, Benzene-*d*_6_) δ 7.07–7.04 (m, 4H, *m*-C_6_*H*_3_), 6.96 (t_app_, 2H, *p*-C_6_*H*_3_), 4.24 (sept, *J* = 6.8 Hz, 2H, C*H*Me_2_), 4.19 (sept, *J* = 6.8 Hz, 2H, C*H*Me_2_), 3.43 (d, ^4^*J*_CH_ = 4.0 Hz, 3H, AlO_2_C(O*Me*)), 1.42 (d, *J* = 6.8 Hz, 6H, CH*Me*_2_), 1.40 (s, 2H, SiC*H*_2_)*, 1.39 (d, *J* = 6.8 Hz, 6H, CH*Me*_2_)*, *overlapping peaks 1.31 (s, 2H, SiC*H*_2_), 1.22 (d, *J* = 6.8 Hz, 6H, CH*Me*_2_), 1.11 (d, *J* = 6.8 Hz, 6H, CH*Me*_2_), 0.71 (s, 12H, BOCC*Me*_2_), 0.43 (s, 6H, Si*Me*_2_), 0.31 (s, 6H, Si*Me*_2_). ^13^C NMR (101 MHz, 298 K, Benzene-*d*_6_) δ 167.0 (*o*-*C*_6_H_3_), 156.4 (MeO*C*O_2_), 155.2 (*o*-*C*_6_H_3_), 148.1 (*i*-*C*_6_H_3_), 123.3 (*m*-*C*_6_H_3_), 123.0 (*m*-*C*_6_H_3_), 121.6 (*p*-*C*_6_H_3_), 80.3 (BO*C*Me_2_), 53.4 (*Me*OCO_2_), 27.0 (BOC*Me*_2_), 25.9 (CH*Me*_2_), 25.6 (*C*HMe_2_), 25.5 (*C*HMe_2_), 24.9 (CH*Me*_2_), 23.5 (CH*Me*_2_), 22.2 (CH*Me*_2_), 14.6 (Si*C*H_2_), 2.2 (Si*Me*_2_), 1.0 (Si*Me*_2_). ^11^B NMR (128 MHz, 298 K, Benzene-*d*_6_) δ 33.8. ^+^MeOBpin impurity was observed.

### Synthesis of Compound **20**

In a J Young’s tube, [K{Al(SiN^Dipp^)(OMe)(B(OMe)_2_)}] (**17**, 35 mg, 0.053 mmol) was dissolved in 0.4 mL of *d*_6_-benzene before the addition of Me_3_SiOTf (9.6 μL, 11.7 mg, 0.053 mmol) *via* a micropipette into the colorless solution. Upon addition, the reaction mixture provided colorless precipitates and a colorless supernatant. The reaction mixture was then left standing at ambient temperature for 2 h before filtration, and the residual solids were further extracted with toluene (0.2 mL × 2). All of the filtrate was then collected and kept under vacuum to remove all volatiles to give **20** as a colorless waxy solid. Yield 25 mg, 68%. Colorless single crystals suitable for X-ray crystallography were obtained by slow evaporation of a toluene/hexane solution (ca. 0.1 mL toluene/2 mL hexane) at ambient temperature. No meaningful result for elemental analysis could be obtained after multiple attempts. ^1^H NMR (400 MHz, 298 K, Benzene-*d*_6_) δ 7.10–6.96 (m, 6H, C_6_*H*_3_), 3.78 (sept, *J* = 6.9 Hz, 4H, C*H*Me_2_), 2.78 (s, 6H, BO*Me*_2_), 1.30 (d, *J* = 6.9 Hz, 12H, CH*Me*_2_), 1.27 (d, *J* = 6.9 Hz, 12H, CH*Me*_2_), 1.12 (s, 4H, SiC*H*_2_), 0.20 (s, 12H, Si*Me*_2_). ^13^C NMR (101 MHz, 298 K, Benzene-*d*_6_) δ 146.4 (*o*-*C*_6_H_3_), 143.0 (*i*-*C*_6_H_3_), 124.4 (*m*-*C*_6_H_3_), 123.8 (*p*-*C*_6_H_3_), 51.4 (BO*Me*_2_), 28.3 (C*H*Me_2_), 24.9 (CH*Me*_2_), 24.7 (CH*Me*_2_), 13.5 (Si*C*H2), −0.9 (Si*Me*_2_). ^11^B NMR (128 MHz, 298 K, Benzene-*d*_6_) δ 34.8.

## Supporting Information Available

NMR spectra and details of the single-crystal X-ray diffraction analysis (PDF)
