# Supplementary material for: Aluminum–Boron Bond Formation by Boron Ester Oxidative Addition at an Alumanyl Anion
Source: Inorg Chem. 2023 Sep 6;62(37):15310–9. doi: 10.1021/acs.inorgchem.3c02566 (PMC10521018; doi:10.1021/acs.inorgchem.3c02566)
Supplement: Supplementary file 1 — ic3c02566_si_001.pdf [file ic3c02566_si_001.pdf]

Supporting Information for:

Aluminum-Boron Bond Formation by Boron Ester Oxidative Addition at an Alumanyl Anion

Han-Ying Liu, Mary F. Mahon and Michael S. Hill*

Department of Chemistry, University of Bath, Claverton Down, Bath, BA2 7AY, UK

NMR spectra: Figure S1: ^{1}H NMR (400 MHz, 298 K, d_6-benzene) spectrum of **15.**

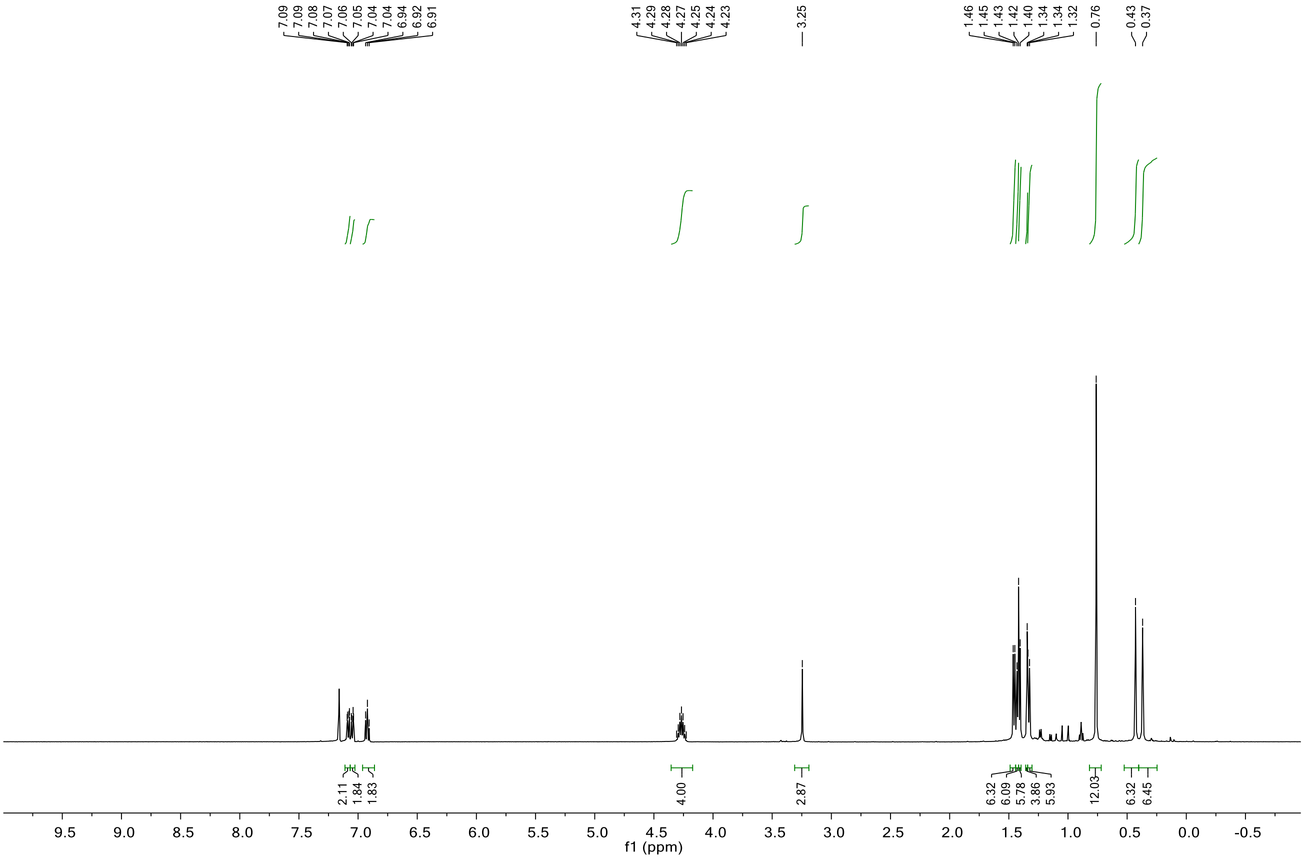

Figure S2: ^{13}C{^{1}H} NMR (101 MHz, 298 K, d_6-benzene) spectrum of **15**.

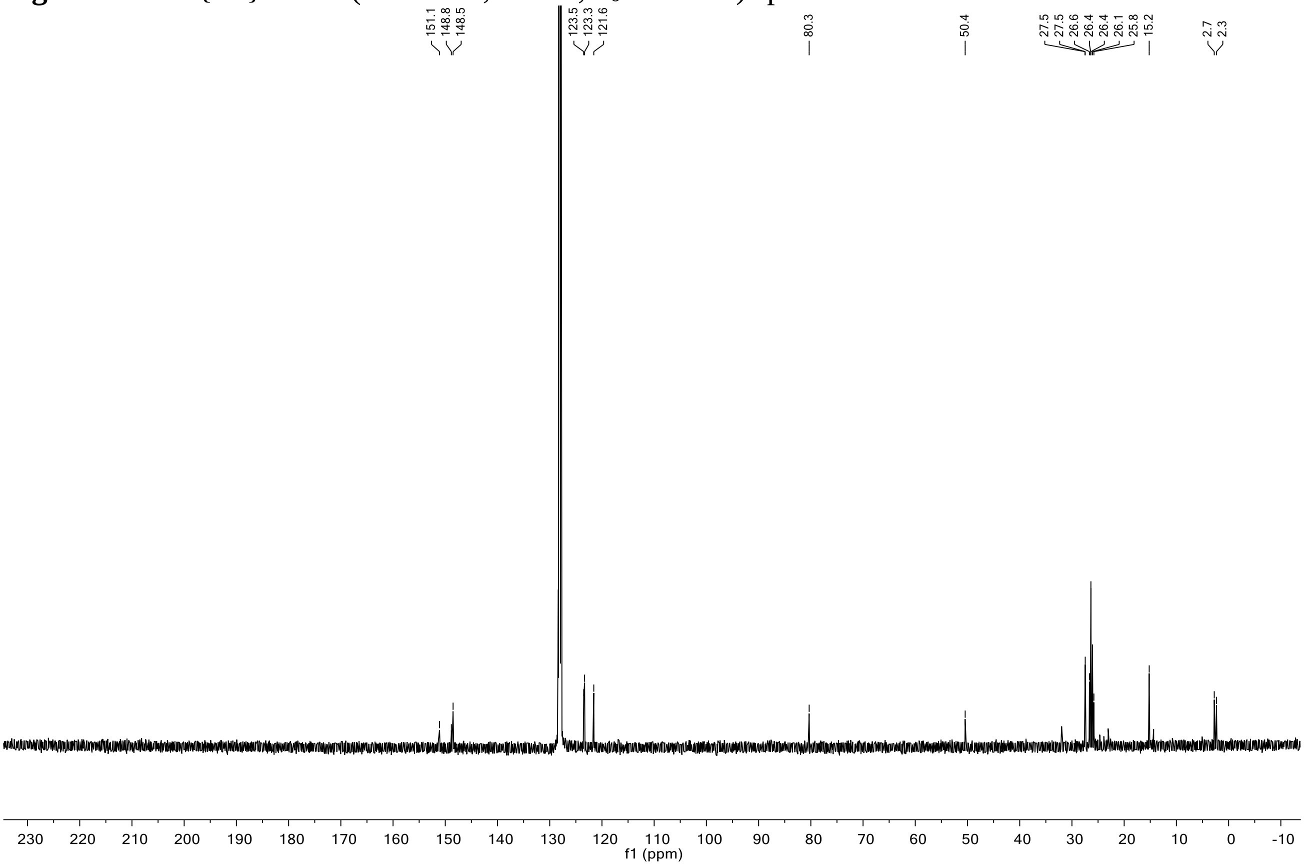

Figure S3: ^{1}H-^{13}C HSQC spectrum of **15**.

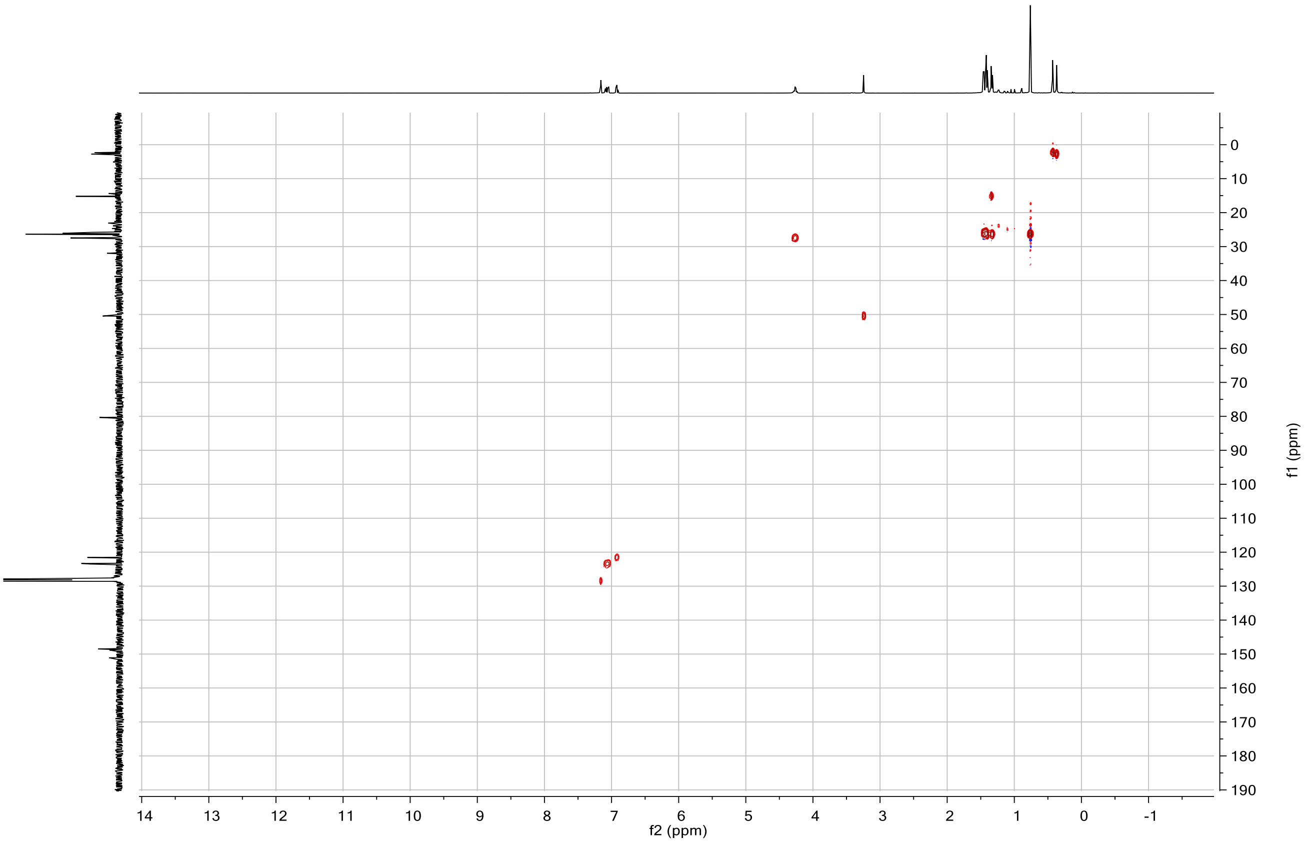

Figure S4: ^{1}H-^{13}C HMBC spectrum of **15**.

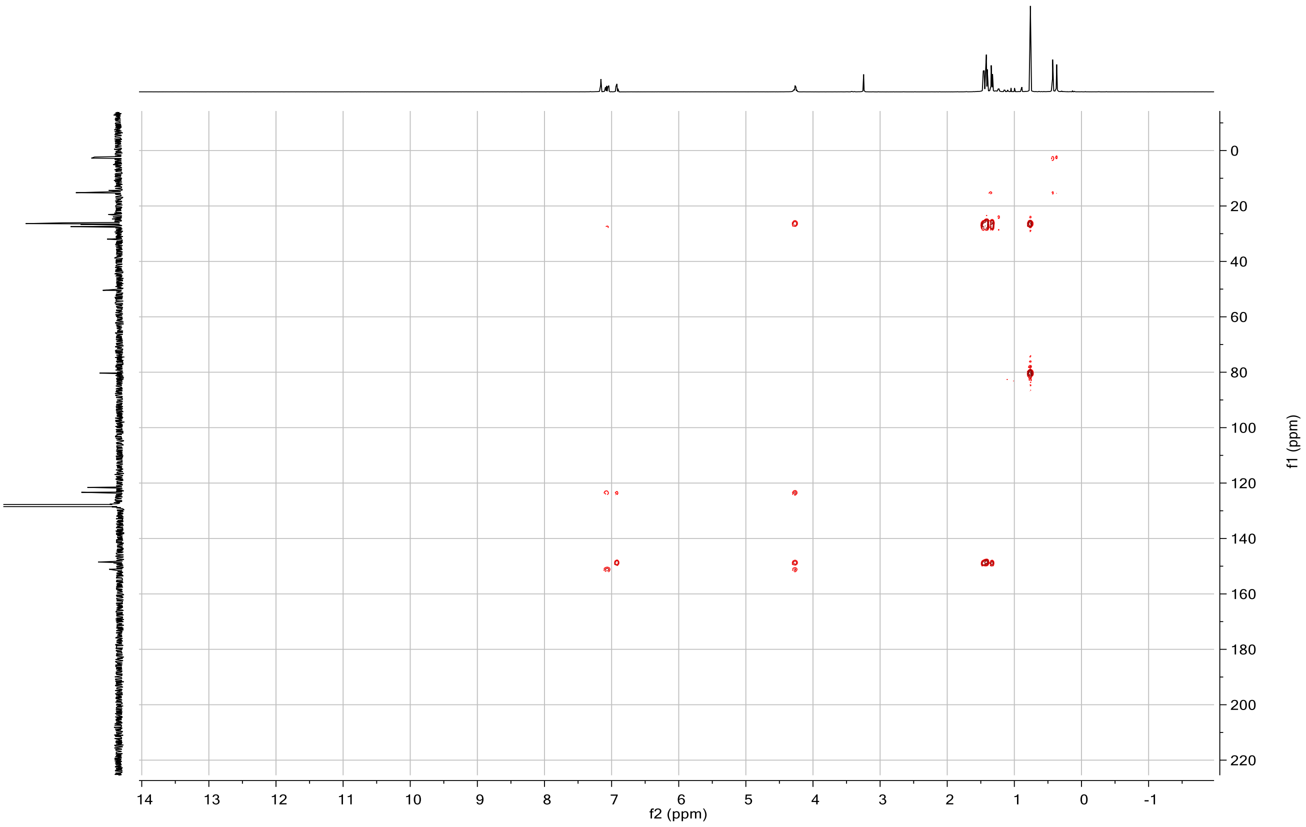

Figure S5: $^{11}B\{^{1}H\}$ NMR (128 MHz, 298 K, d_6-benzene) spectrum of **15.**

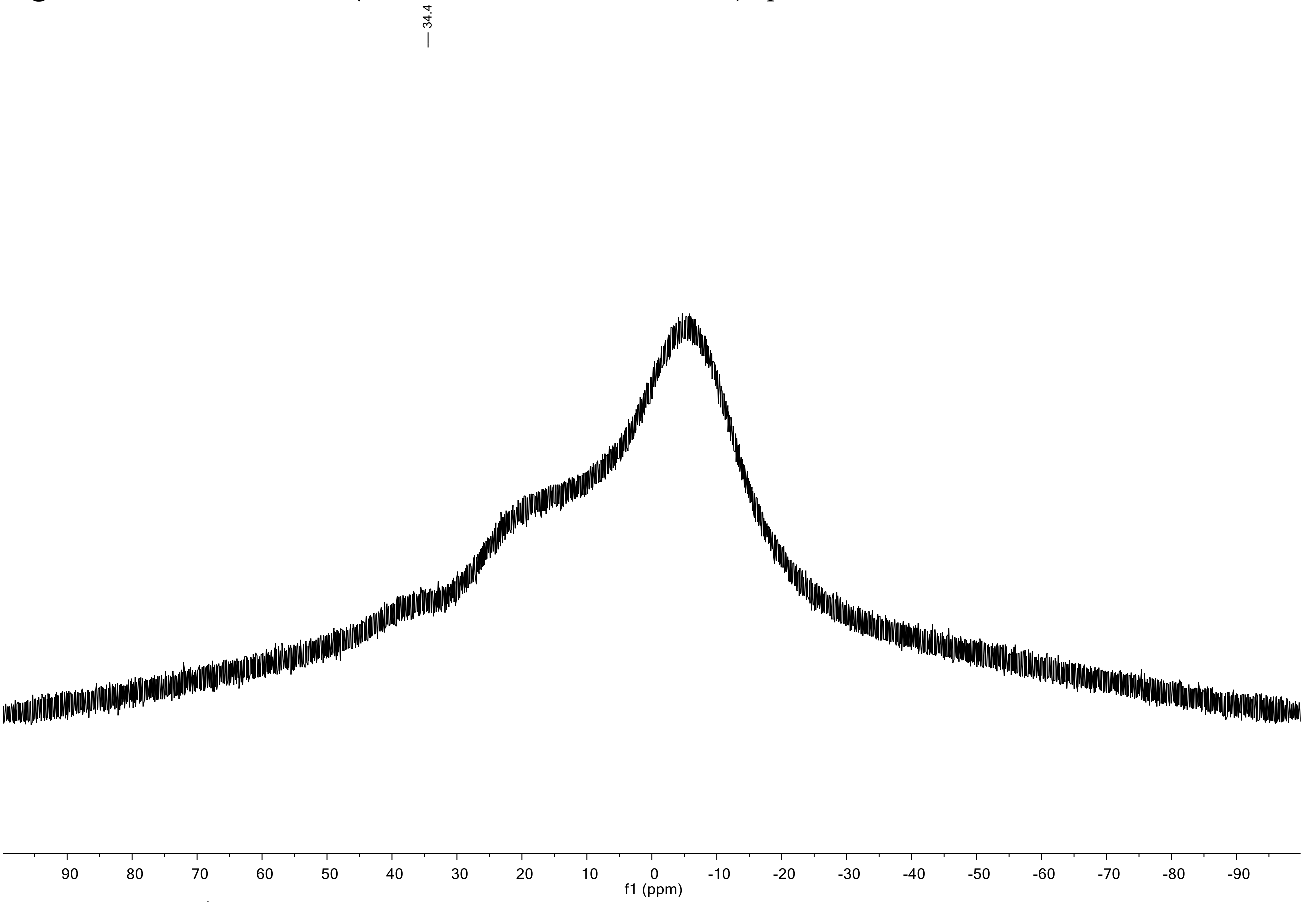

Figure S6: ^{1}H NMR (400 MHz, 298 K, d_6-benzene) spectrum of **16.** *hexane

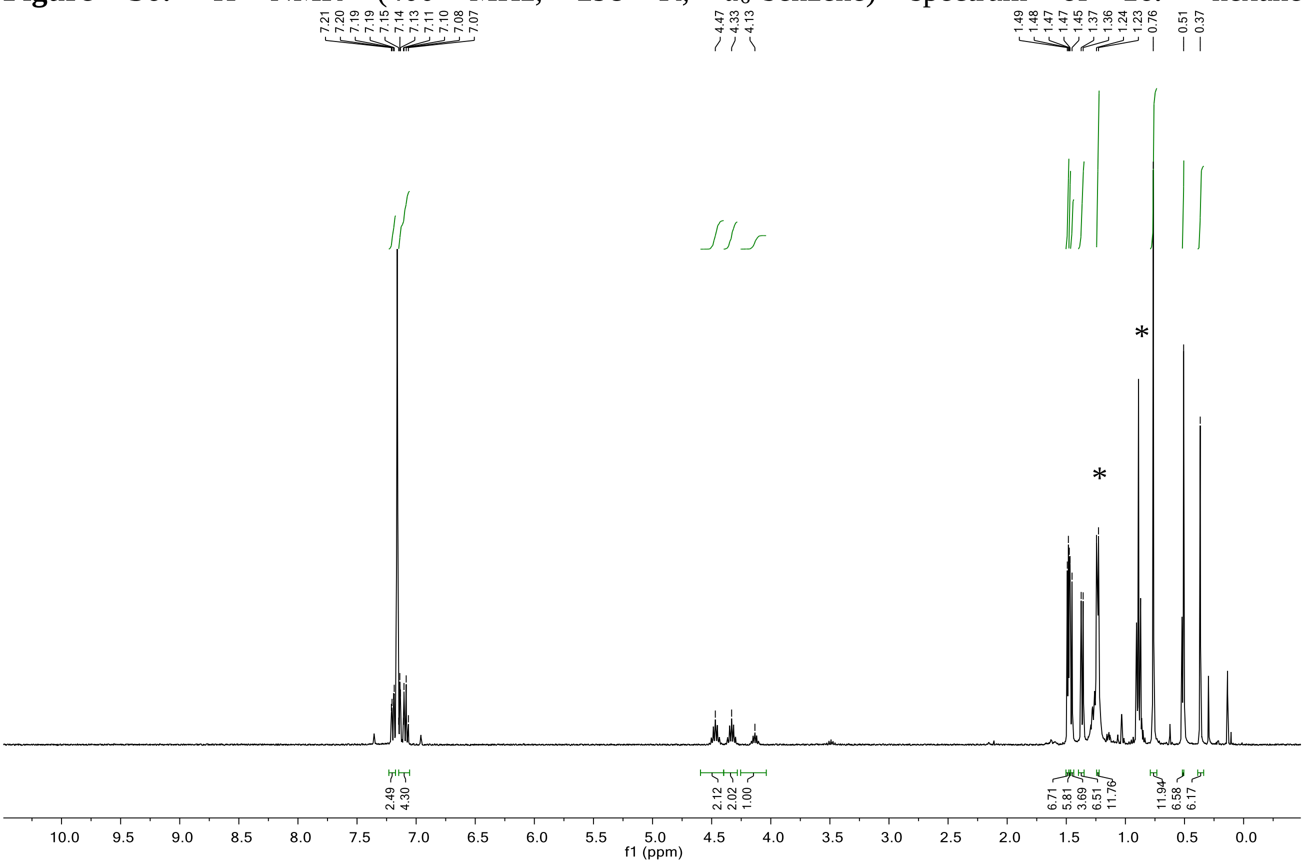

Figure S7: $^{13}C\{^{1}H\}$ NMR (101 MHz, 298 K, d_6-benzene) spectrum of **16**. *hexane

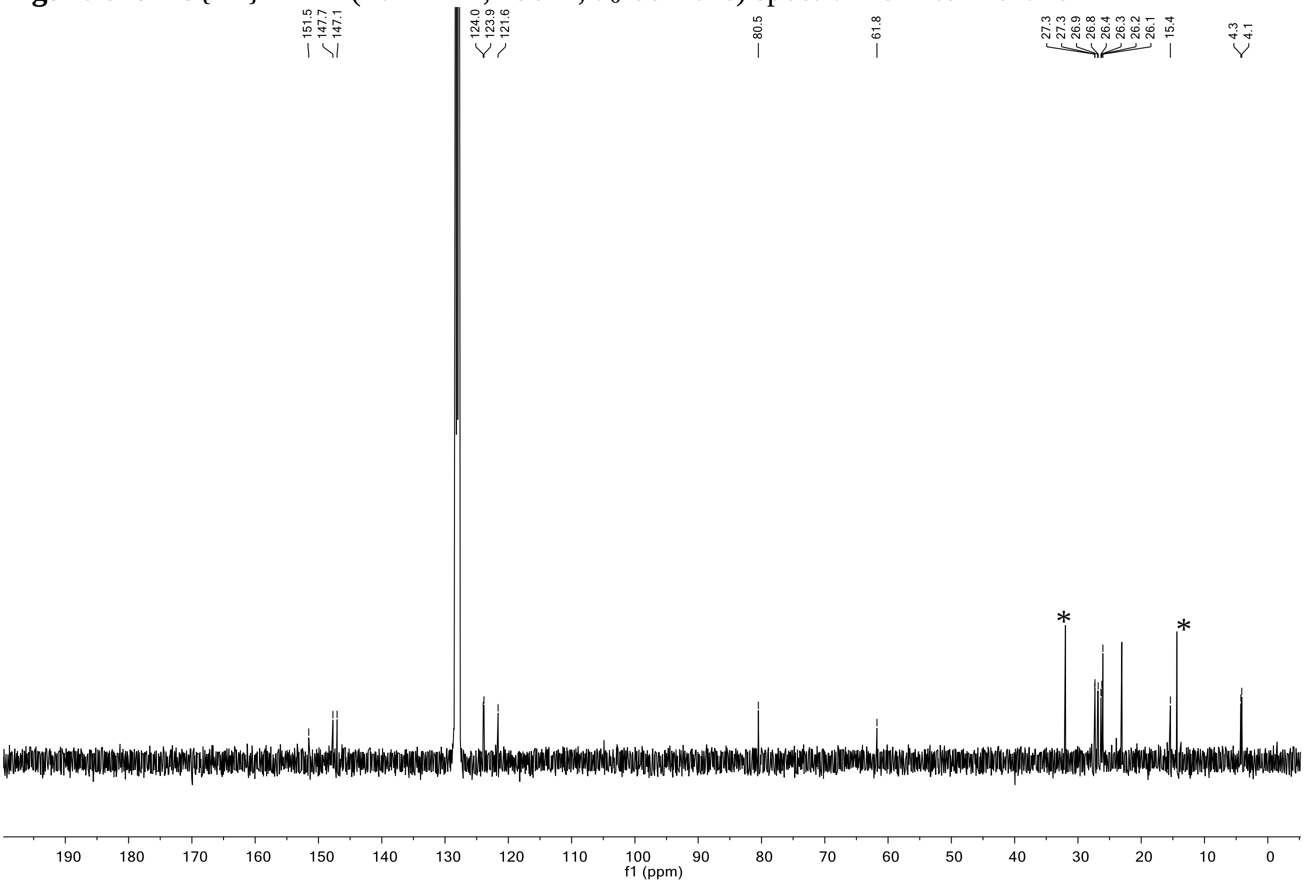

Figure S8: ^{1}H-^{13}C HSQC spectrum of **16**.

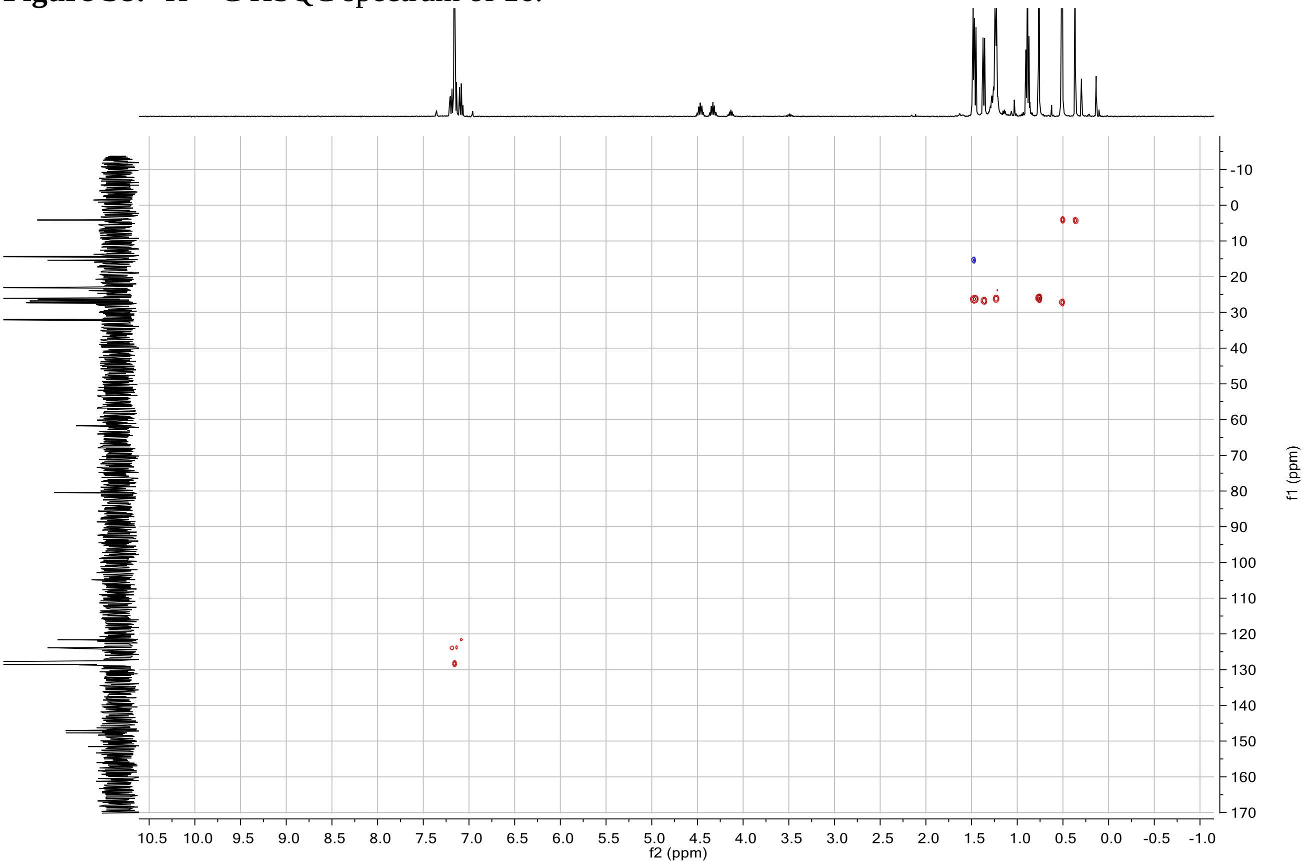

Figure S9: ^{1}H-^{13}C HMBC spectrum of **16**.

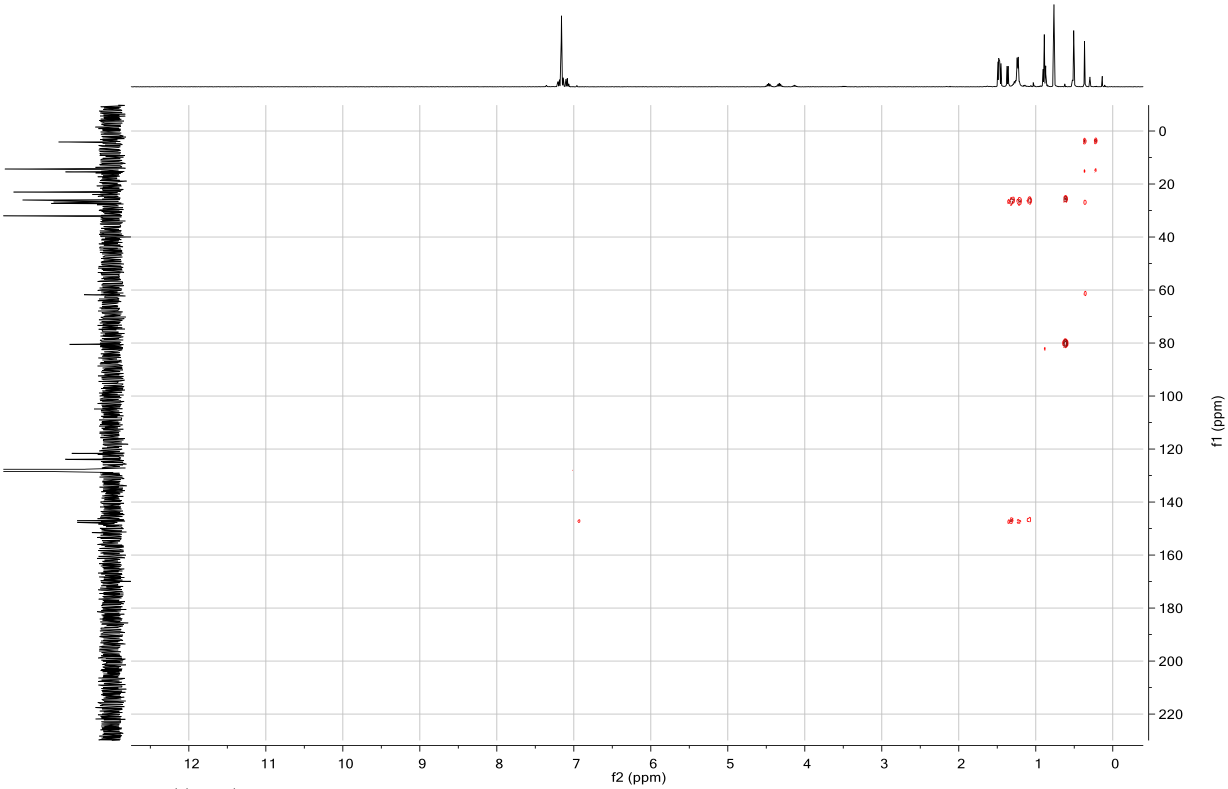

Figure S10: $^{11}B\{^{1}H\}$ NMR (128 MHz, 298 K, d_6-benzene) spectrum of **16.**

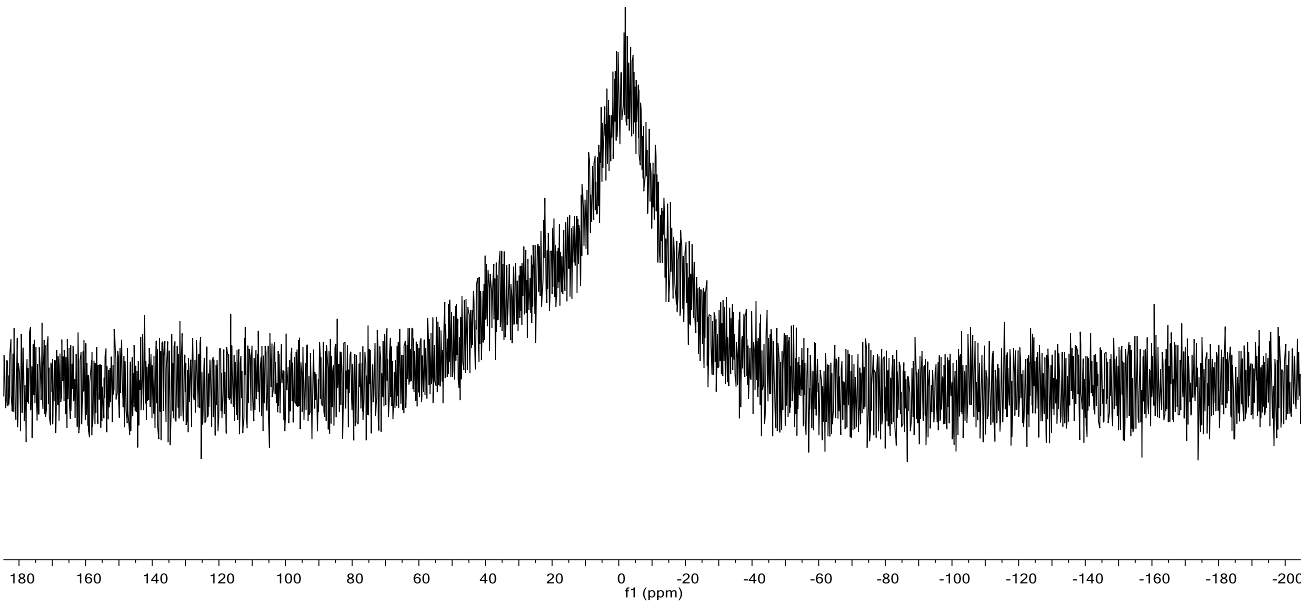

Figure S11: ^{1}H NMR (400 MHz, 298 K, d_6-benzene) spectrum of **17.** *hexane

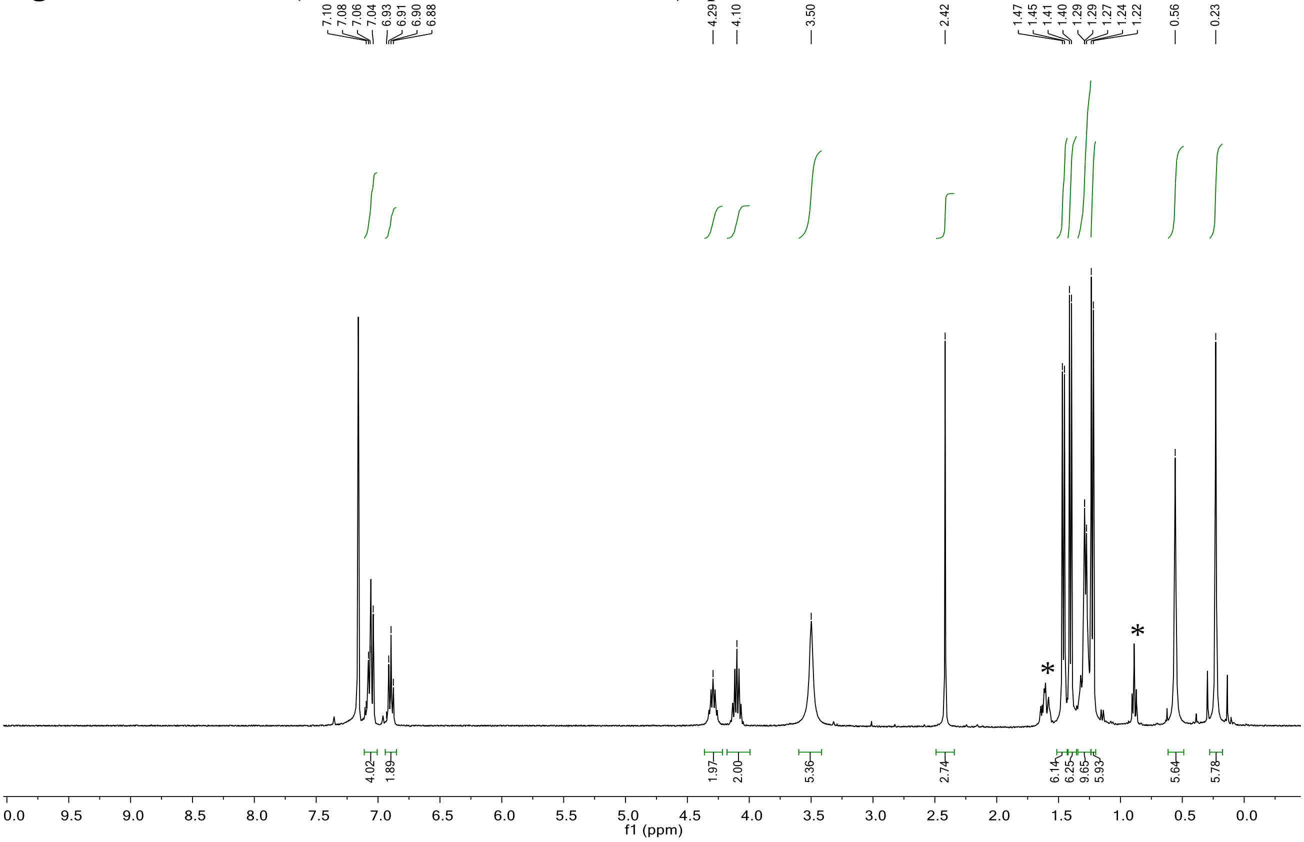

Figure S12: $^{13}C\{^{1}H\}$ NMR (101MHz, 298 K, d_6-benzene) spectrum of **17**. *hexane

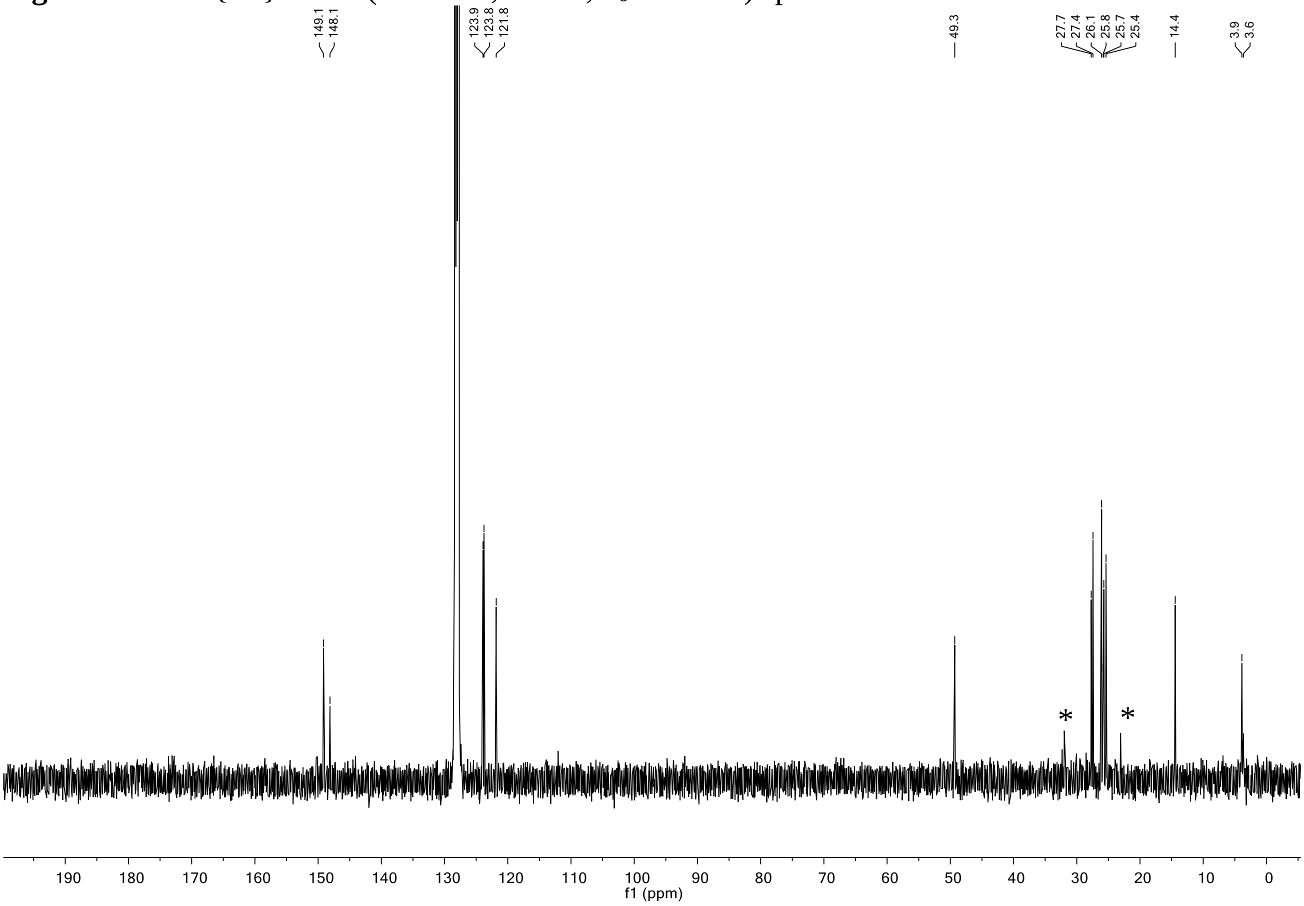

Figure S13: ^{1}H-^{13}C HSQC spectrum of **17.**

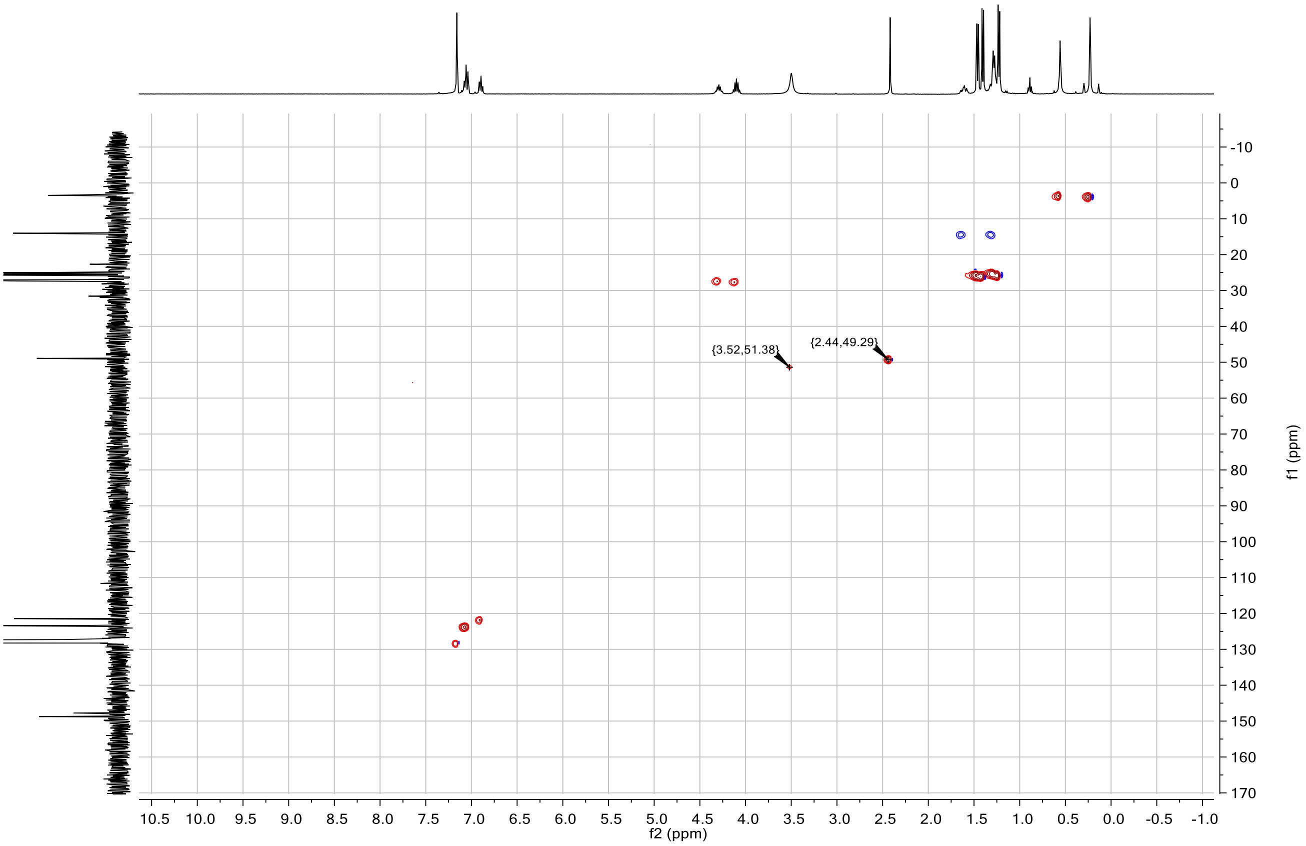

Figure S14: ^{1}H-^{13}C HMBC spectrum of **17**.

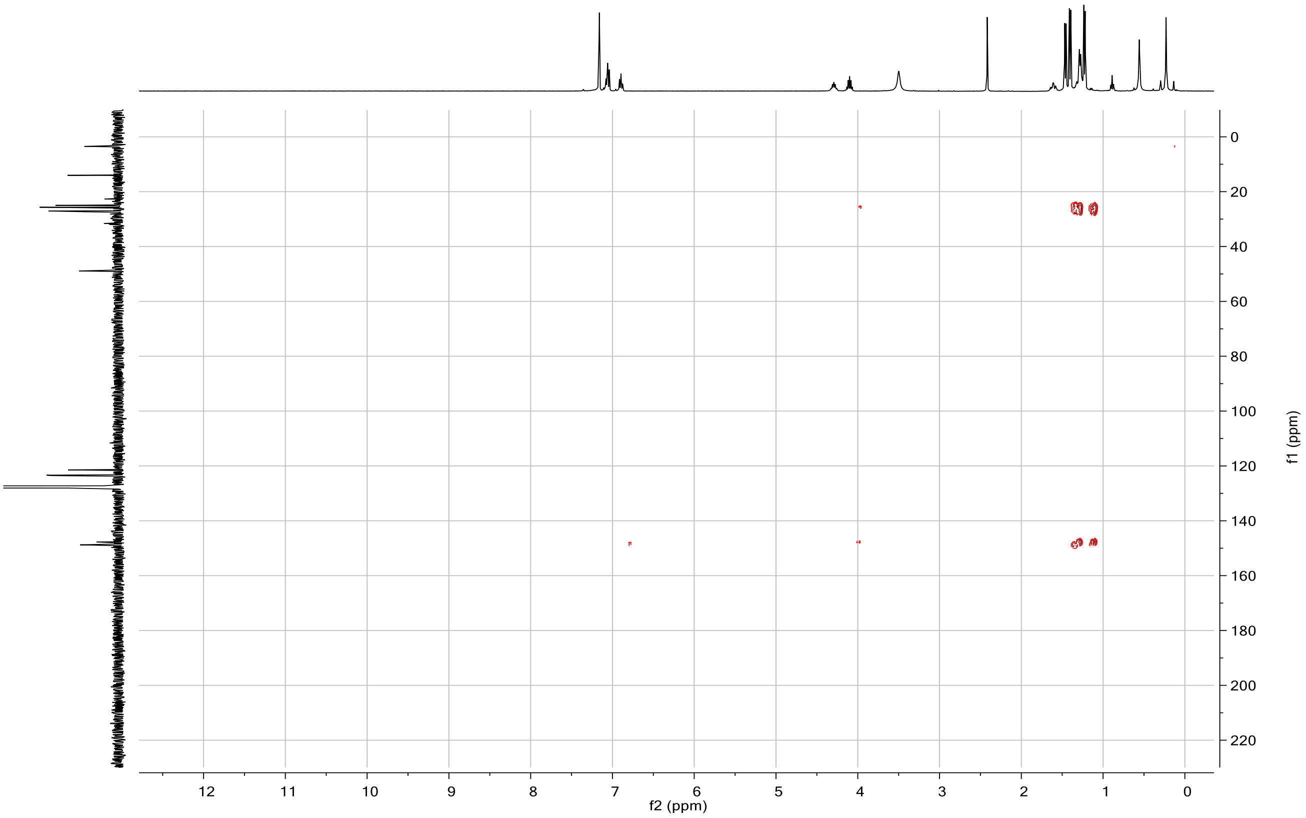

Figure S15 $^{11}B\{^{1}H\}$ NMR (128 MHz, 298 K, d_6-benzene) spectrum of **17.**

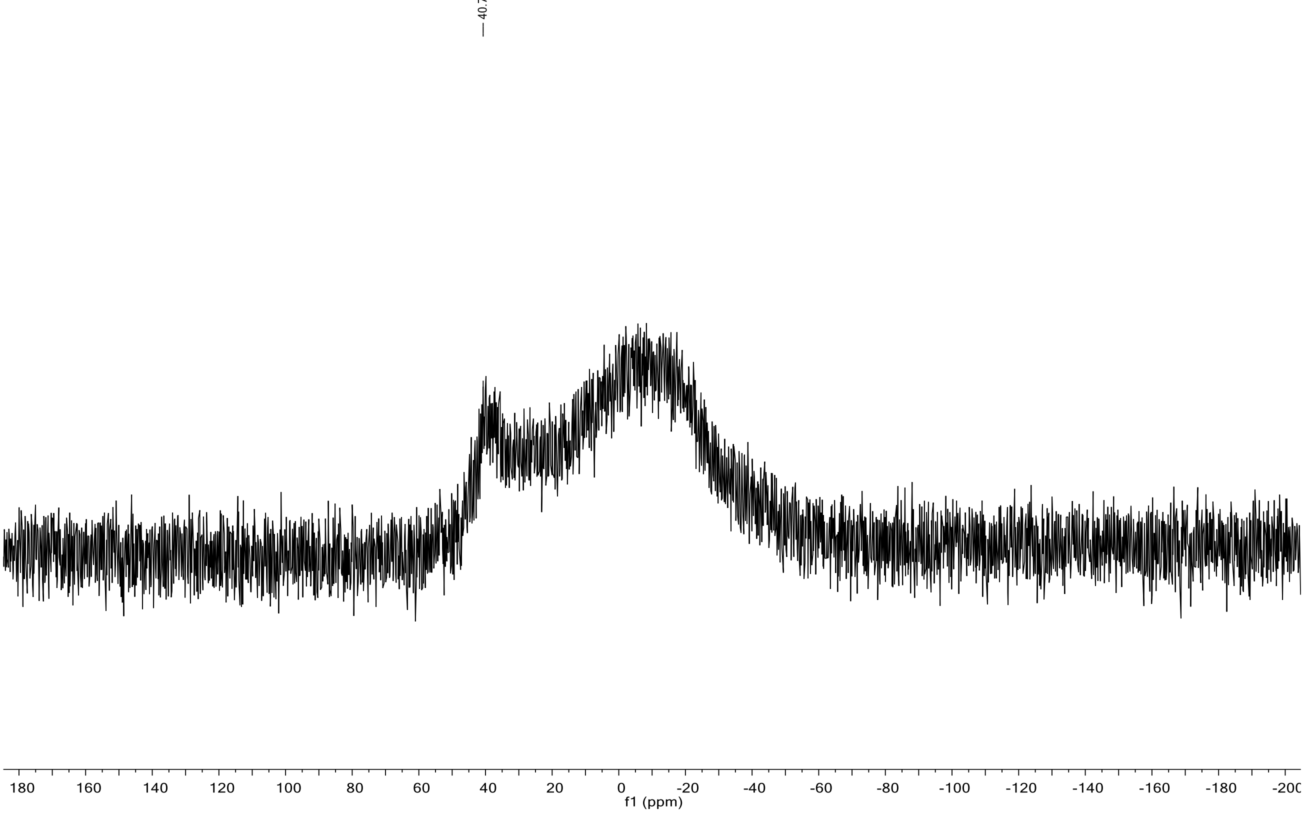

Figure S16: ^{1}H NMR (400 MHz, 298 K, d_6-benzene) spectrum of the reaction mixture of *N*,*N'*-di-isopropylcarbodiimide and **15** after being being kept at 110 °C for one hour.

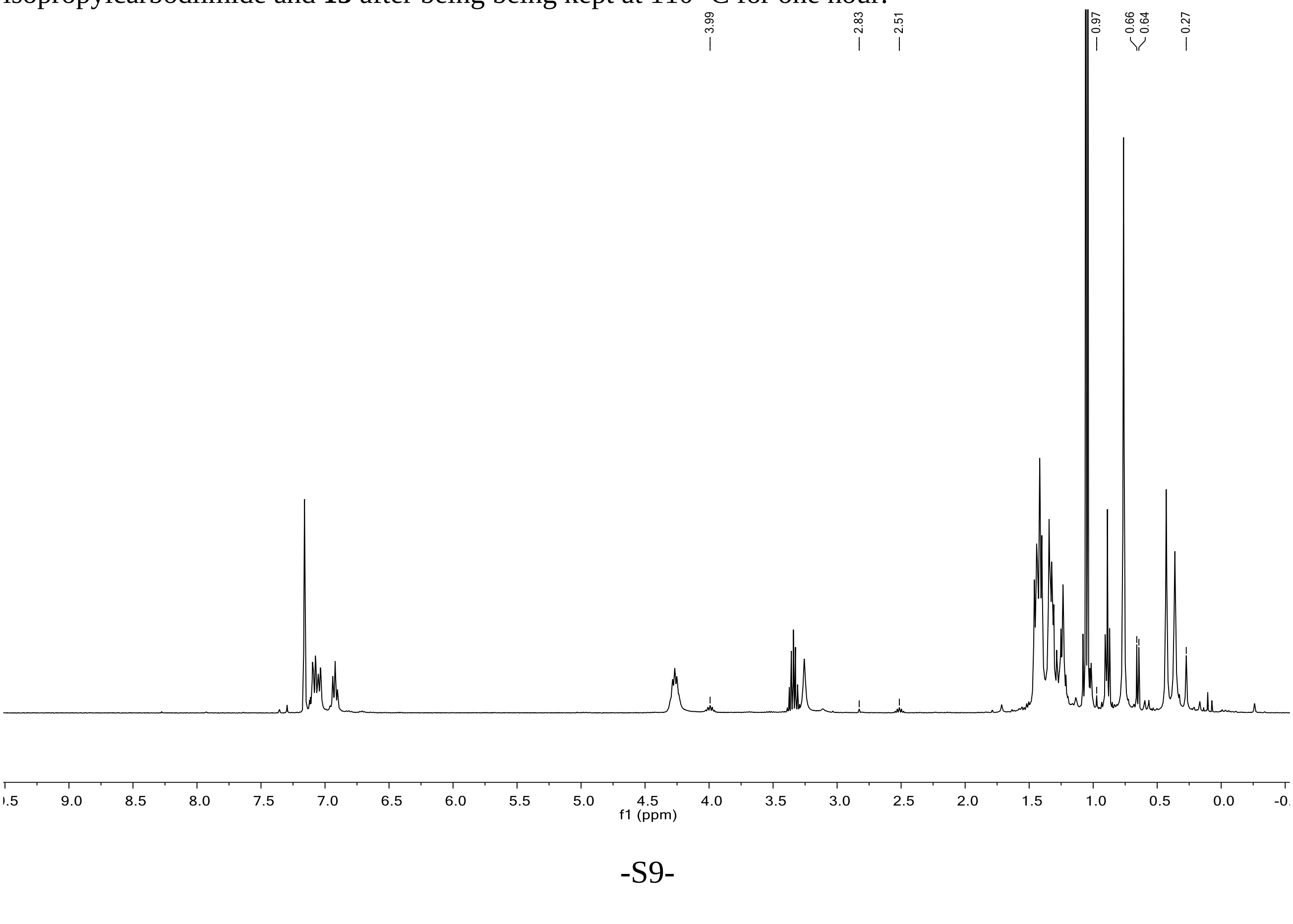

Figure S17: ^{1}H NMR (400 MHz, 298 K, d_6-benzene) spectrum of the reaction mixture of *N*,*N'*-di-isopropylcarbodiimide and **15** after being kept at 110 °C for 16 hours.

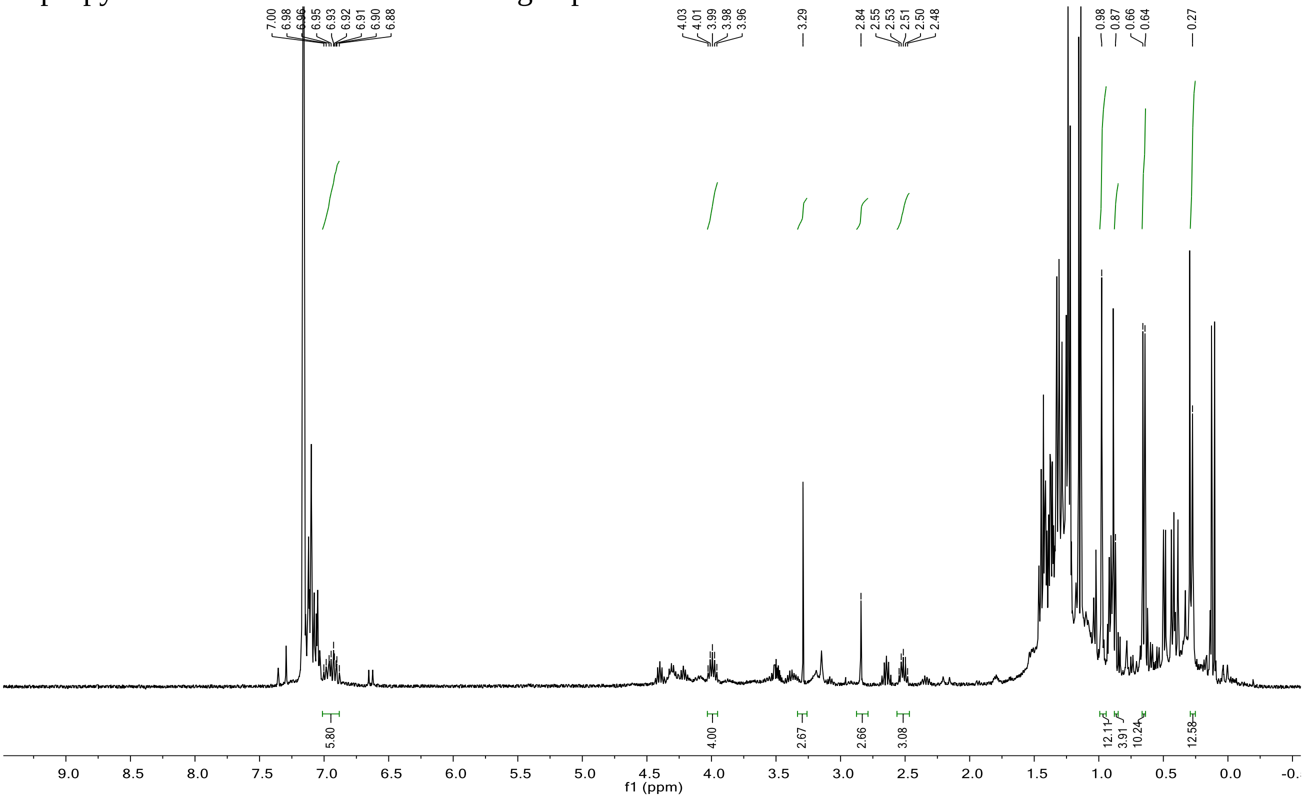

Figure S18: ^{1}H NMR (400 MHz, 298 K, d_6-benzene) spectrum of **19.** *grease, $^+$ MeOBpin.

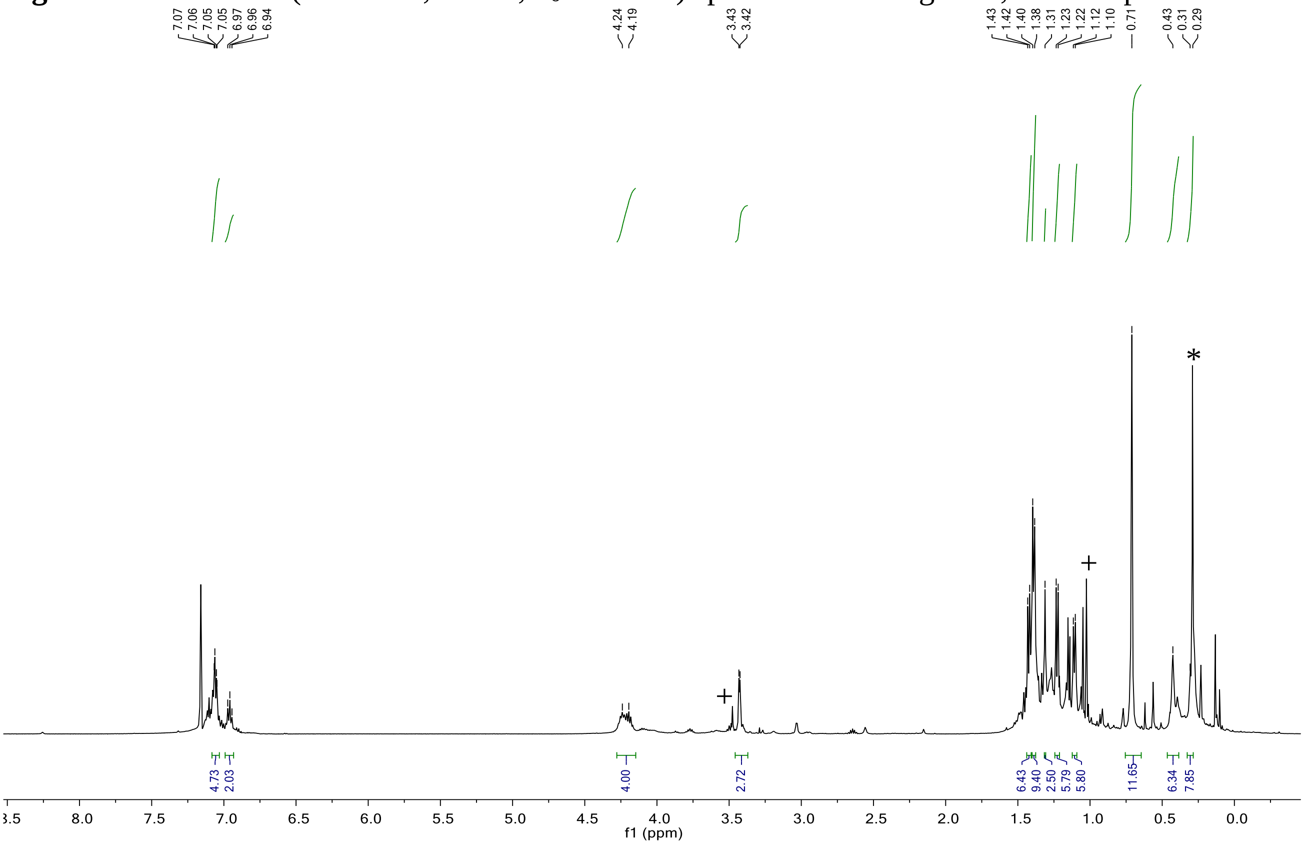

Figure S19: $^{13}C\{^{1}H\}$ NMR (101MHz, 298 K, d_6-benzene) spectrum of **19**.

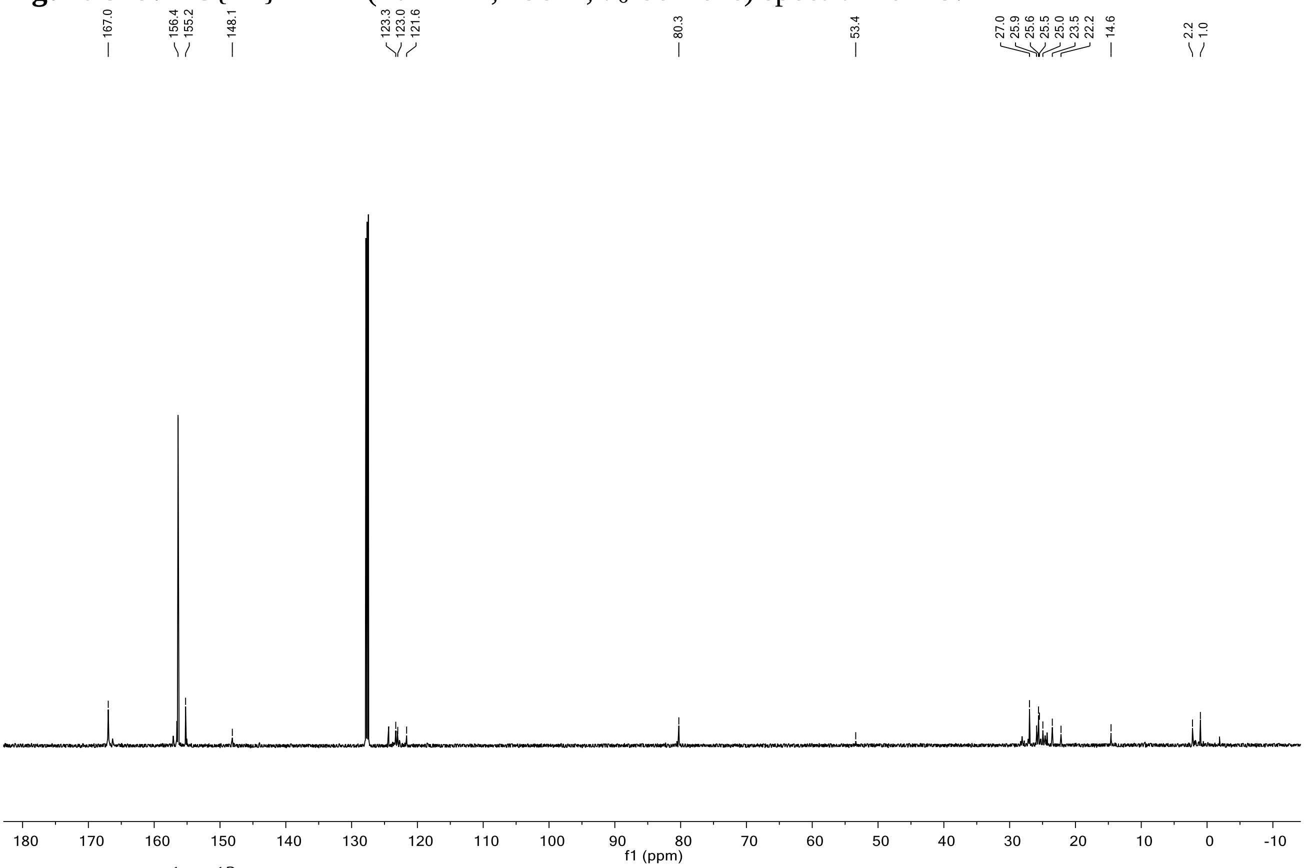

Figure S20: ^{1}H-^{13}C HSQC spectrum of **19**.

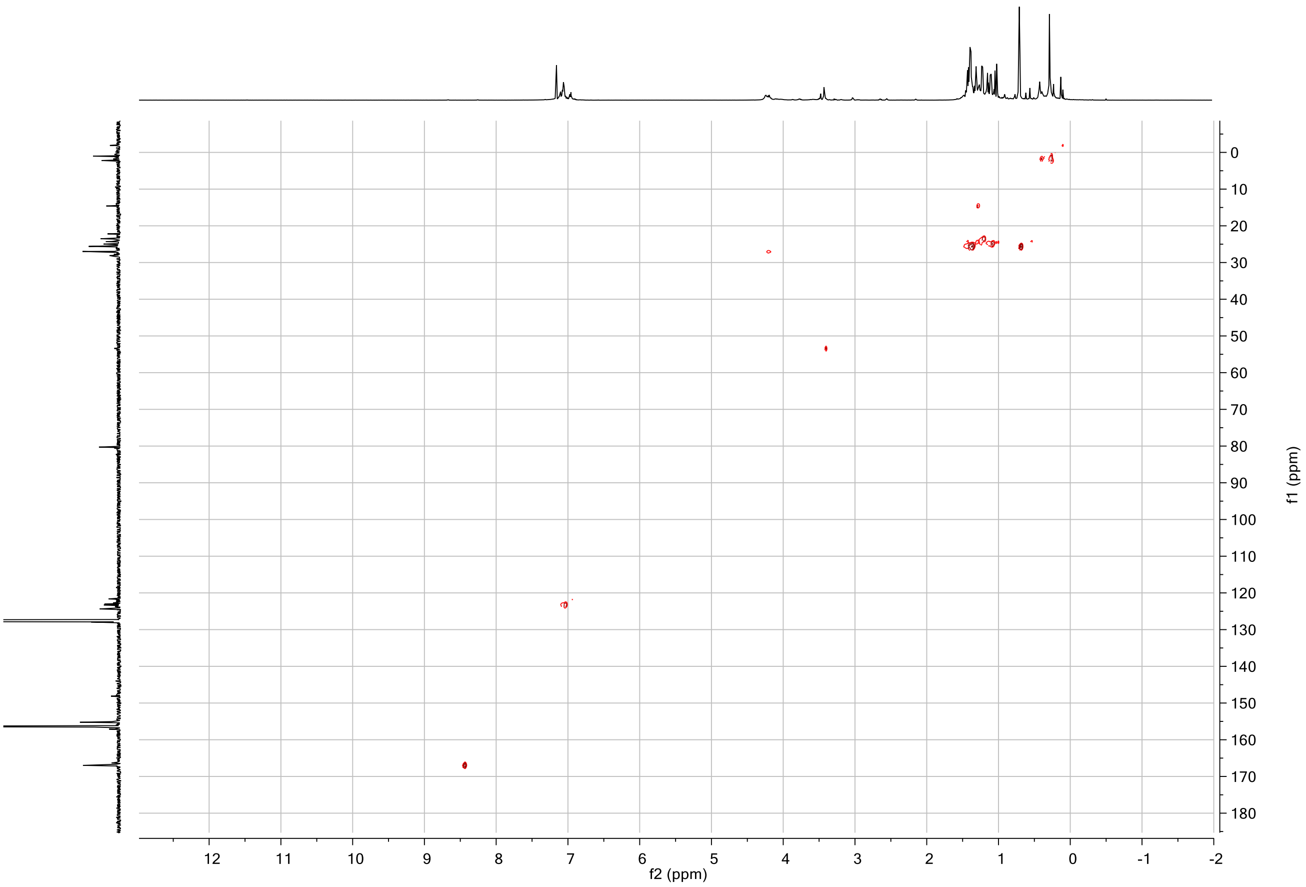

Figure S21: ^{1}H-^{13}C HMBC spectrum of **19**.

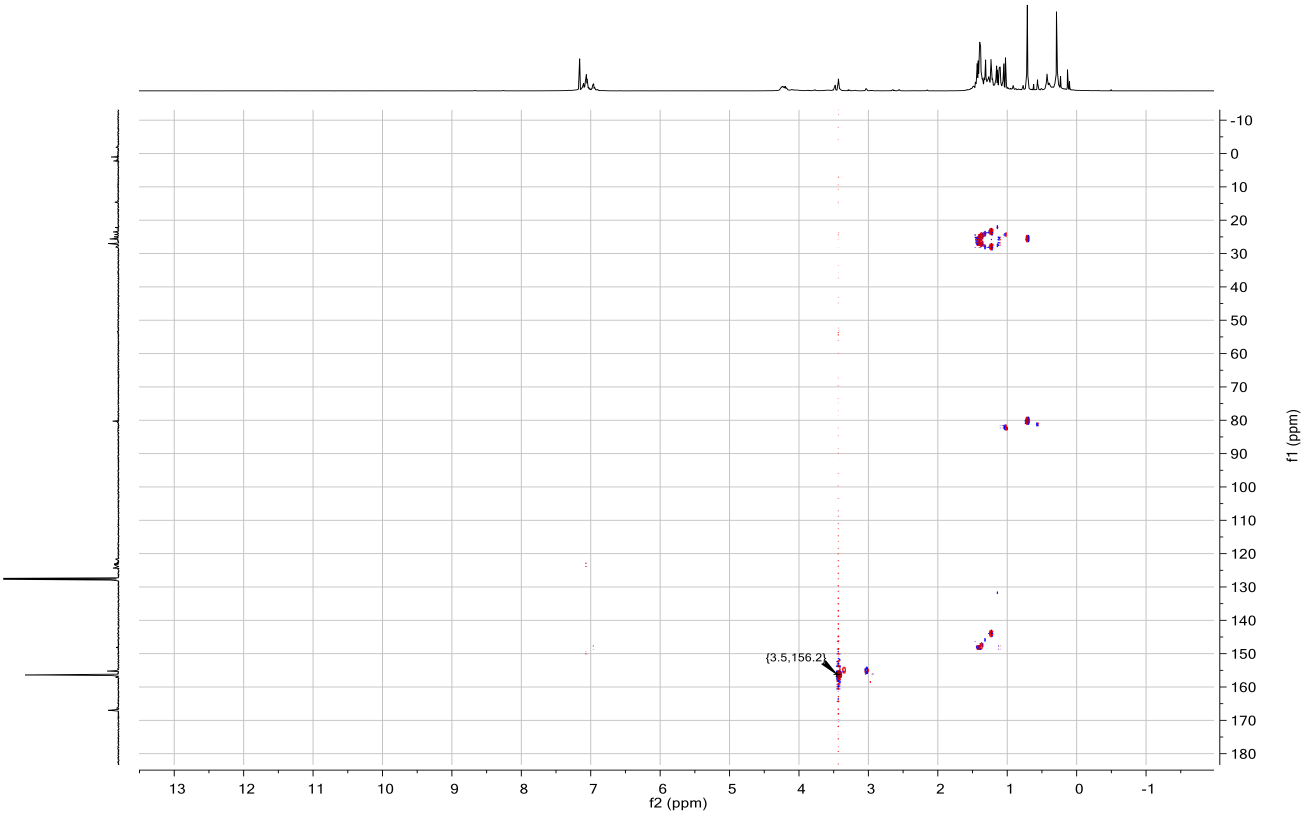

Figure S22: ^{11}B{^{1}H} NMR (128 MHz, 298 K, d_6-benzene) spectrum of **19.** $^{+}$ MeOBpin.

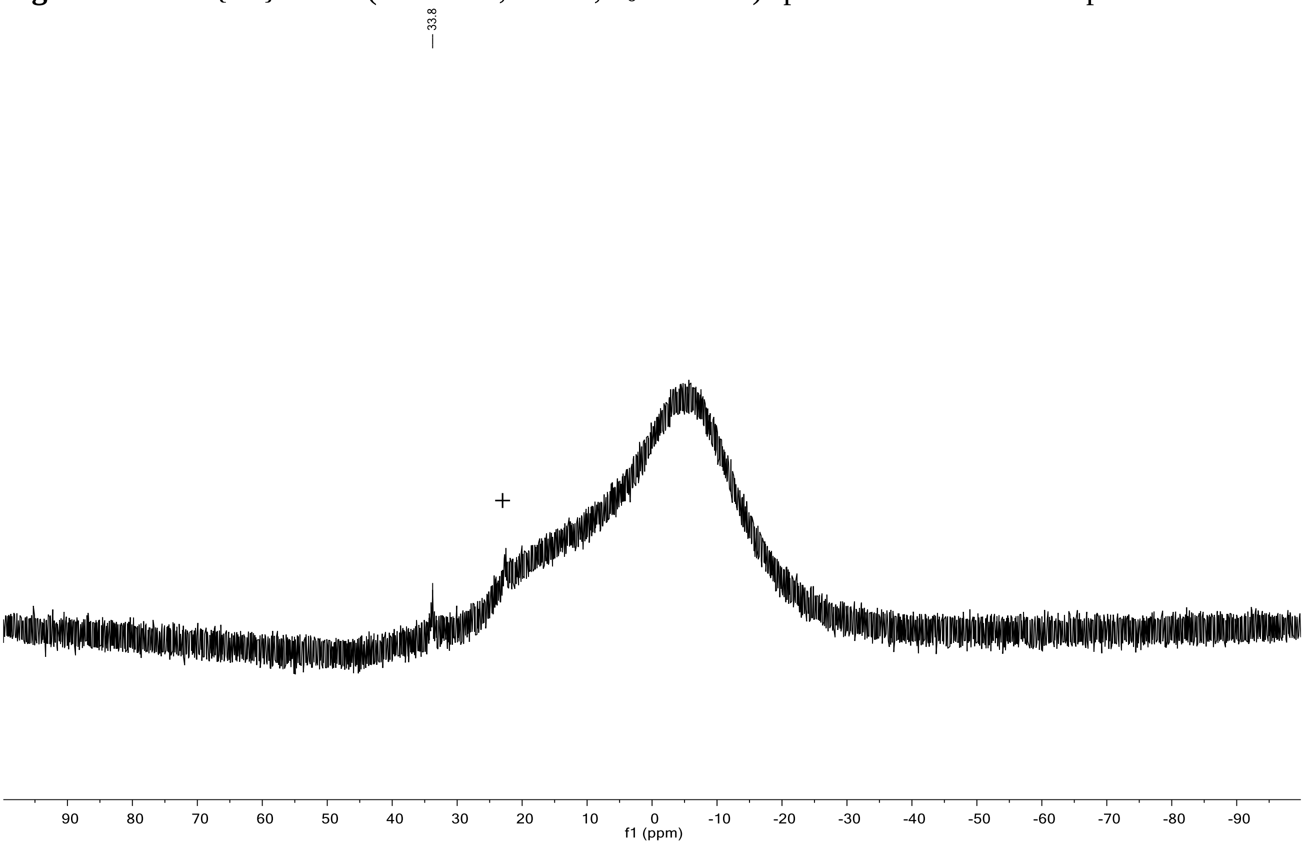

Figure S23: ^{1}H NMR (400 MHz, 298 K, d_6-benzene) spectrum of **20.**

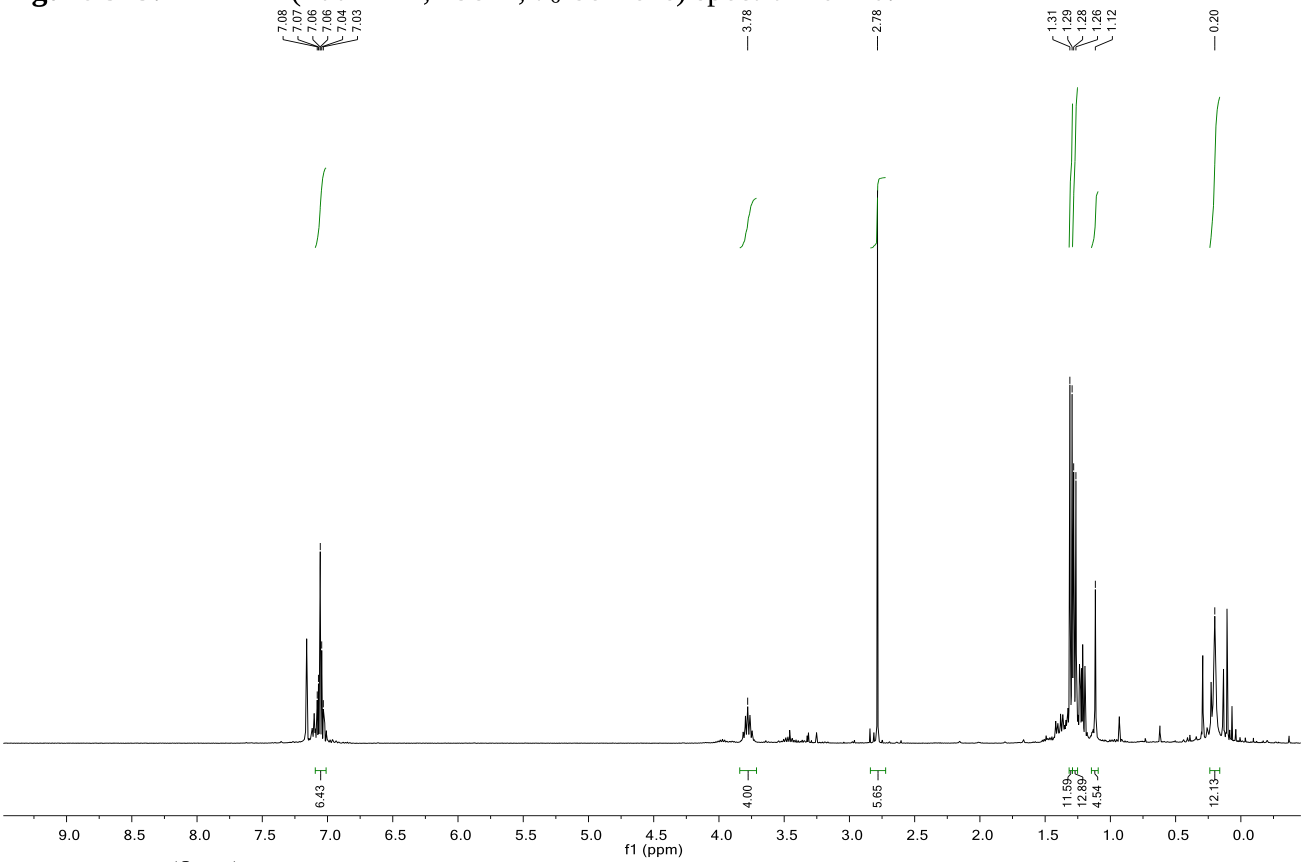

Figure S24: ^{13}C{^{1}H} NMR (101 MHz, 298 K, d_6-benzene) spectrum of **20**. *n-hexane

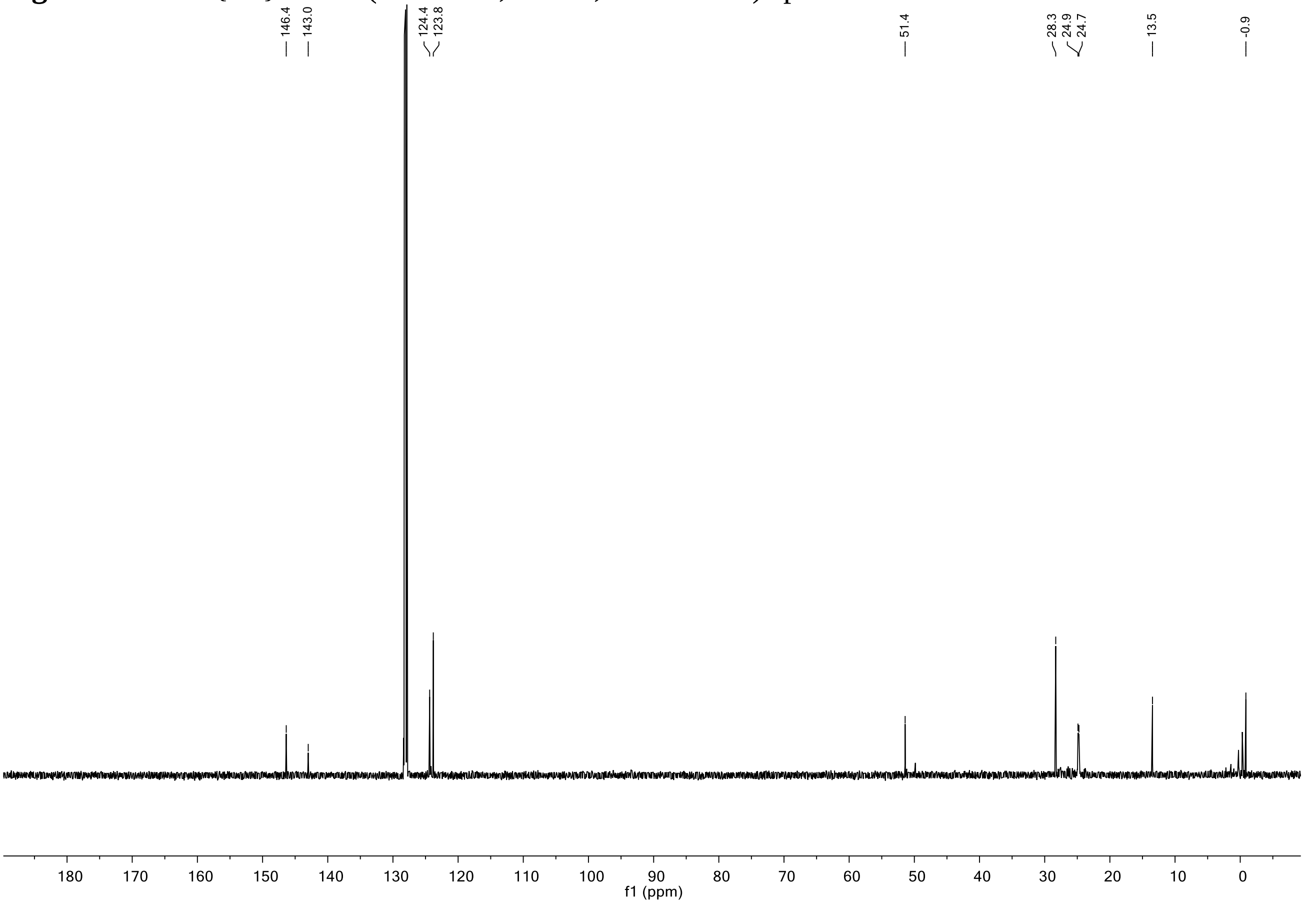

Figure S25 $^{11}B\{^1H\}$ NMR (128 MHz, 298 K, d_6-benzene) spectrum of **20.**

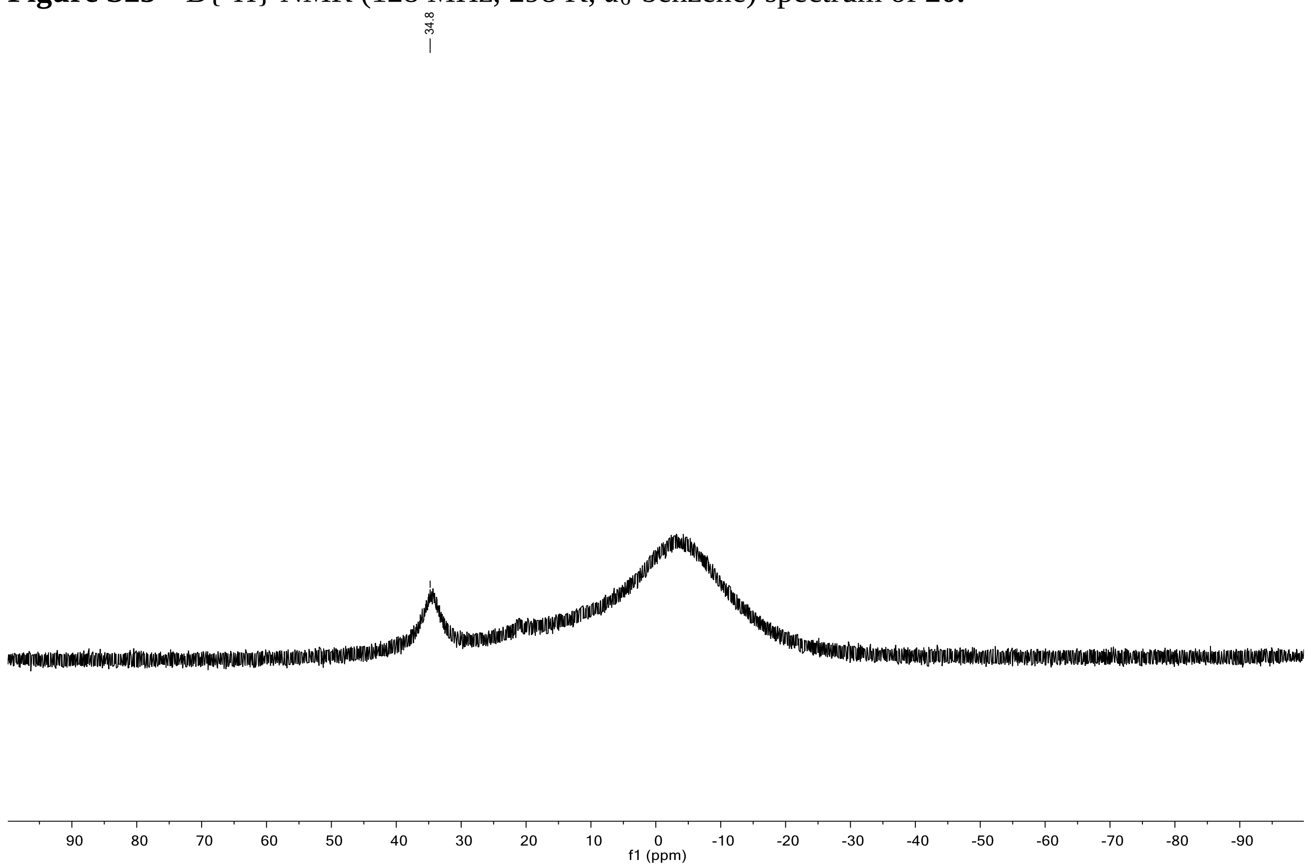

Single Crystal X-ray Diffraction Analysis

Single Crystal X–ray diffraction data for compounds **15** - **20** were collected on a SuperNova EosS2 diffractometer using CuKα (λ = 1.54184 Å) radiation. The crystals were maintained at 150 K during data collection. Using Olex2,[1] the structures were solved with the olex2.solve[2] structure solution program or ShelXT and refined with the ShelXL[3] refinement package using Least Squares minimisation. Noteworthy points follow, and where disorder has been modelled, both distance and ADP restraints have been employed, on merit, in these regions to assist convergence.

In the structure of **15**, the asymmetric unit contains a monomer (which contributes to a dimer the remainder of which is completed via inversion symmetry) plus a region of solvent. While the main feature is entirely ordered, the electron density pertaining to the guest solvent was noted to be very smeared in the direction of the structural channels, along *c*, in which it is located. As such, this was optimally treated using the solvent mask algorithm available in Olex 2, and allowance made for one molecule of hexane, in the motif formula, as presented.

A monomer which propagates to generate 1-D polymers in the gross structure plus half of a molecule of benzene (straddling a crystallographic inversion centre) constitute the asymmetric unit of **16**. The Hydrogen atoms attached to C26, C38 and C38 were located and refined at a distance of 0.98 Å from the relevant parent atom. Disorder (55:45) pertaining to the carbons in the borane ligand was readily modelled, as was 65:35 disorder of the solvent moiety. Distance and ADP restraints were employed, on merit, in each disorder region to assist convergence.

In **17**, the asymmetric unit is constituted by a monomer. This moiety the basis for a gross structure that is dominated by 1-D polymers parallel to the *b* axis. The hydrogen atoms attached to C33 were located and refined freely, subject to being located 0.98 Å from the parent atom.

The asymmetric unit constitutes half of a dimer in the structure of **18**. The remainder of the molecule arises by virtue of crystallographic inversion symmetry. While all hydrogens were included at calculated positions, those with potential contact to the potassium centres were refined with free U_{iso} values in order to afford additional credibility regarding their locations.

The asymmetric unit in **19** contains half of a dimer (the remainder of which arises by virtue of crystallographic inversion symmetry) and one and a half molecules of hexane. Disorder prevailed in both the main feature and in the solvent. In the former, the Bpin carbons were modelled to take account

of a 50:50 split over two proximate sites. The solvent presented as one complete molecule of hexane which did lend itself to some exceedingly restrained disorder treatment and a separate region of extremely smeared electron density. Ultimately, both areas were addressed using the solvent-mask algorithm in Olex-2, and an appropriate allowance made in the formula as presented. The crystal was refined subject to being a racemic twin and this may contribute to the location of the three largest residual electron-density peaks, in a manner that might suggest disorder of O1, O2 and O3. Modelling of this putative disorder was rigorously explored, but it was ultimately abandoned as concomitant disorder of the aluminium and potassium centres (in order to achieve a chemically sensible model) did not prove accessible. In trials, the maximum level of oxygen disorder refined to approximately 10% and if this was credible, one would expect observation of a minor component for both Al1 and K1! Otherwise, this experiment has rendered a very credible structural characterisation of this material.

There are two molecules present in the asymmetric unit of **20**. The largest residual electron density peak is an artefact, and most likely reflects the presence of a small passenger on the main crystal. A twin integration only served to degrade the data, because of the very low diffracting ability of the second component. As such, the model presented herein is based on a data reduction that recognises only the major sample component. Refinement has also taken account of racemic twinning in this crystal structure.

Figure S26: Space filling representation (Mercury) of the structure of compound **20**, illustrating the steric protection afforded the aluminum (pink) to boron (yellow) bond.

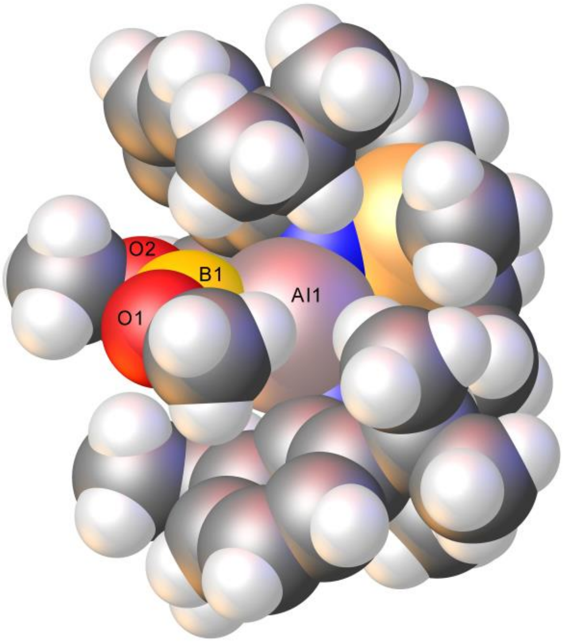

Table S1: Crystal data and structure refinement for compounds **15**, **16**, **17** and **18**.

Identification code	**15**	**16**	**17**	**18**
Empirical formula	$C_{43}H_{79}AlBKN_2O_3Si_2$	$C_{42}H_{72}AlBKN_2O_3Si_2$	$C_{33}H_{59}AlBKN_2O_3Si_2$	$C_{49}H_{89}AlBK_2N_5O_4Si_2$
Formula weight	805.15	786.08	664.89	984.42
Crystal system	trigonal	monoclinic	monoclinic	triclinic
Space group	*R*-3	$P2_1/c$	$P2_1/c$	*P*-1
a/ Å	45.1022(7)	20.6465(1)	11.93610(10)	11.4414(2)
b/ Å	45.1022(7)	13.1305(1)	18.86170(10)	13.2734(2)
c/ Å	12.4356(2)	18.1611(1)	16.85920(10)	18.5530(3)
α/ °	90	90	90	93.257(1)
β/ °	90	111.896(1)	95.9530(10)	90.496(1)
γ/ °	120	90	90	91.750(1)
U/ Å^3	21907.5(8)	4568.29(6)	3775.13(4)	2811.56(8)
Z	18	4	4	2
ρ_{calc}/ g cm^{-3}	1.099	1.143	1.170	1.163
μ/ mm^{-1}	1.871	1.986	2.317	2.386
F(000)	7920.0	1708.0	1440.0	1068.0
Crystal size/ mm^3	0.122 × 0.095 × 0.076	0.204 × 0.15 × 0.132	0.207 × 0.117 × 0.066	0.121 × 0.101 × 0.068
2θ range for data collection/°	7.46 to 146.258	8.164 to 145.768	8.802 to 145.9	7.732 to 145.844
Index ranges	$-49 \le h \le 55$<br>$-55 \le k \le 53$<br>$-15 \le l \le 15$	$-25 \le h \le 25$<br>$-16 \le k \le 16$<br>$-21 \le l \le 22$	$-14 \le h \le 14$<br>$-23 \le k \le 23$<br>$-20 \le l \le 17$	$-14 \le h \le 14$<br>$-16 \le k \le 16$<br>$-22 \le l \le 21$
Reflections collected	78287	60004	50720	56239
Independent reflections, R_{int}	9706, 0.0489	9101, 0.0366	7530, 0.0410	11153 ,0.0364
Data/restraints/parameters	9706/0/444	9101/230/620	7530/3/414	11153/0/607
Goodness–of–fit on F^2	1.052	1.043	1.026	1.038
Final *R*1, *wR*2 [I>=2σ(I)]	R_1 = 0.0395, wR_2 = 0.1043	R_1 = 0.0357, wR_2 = 0.0969	R_1 = 0.0335, wR_2 = 0.0899	R_1 = 0.0369, wR_2 = 0.1023
Final *R*1, *wR*2 [all data]	R_1 = 0.0469, wR_2 = 0.1092	R_1 = 0.0383, wR_2 = 0.0993	R_1 = 0.0378, wR_2 = 0.0936	R_1 = 0.0394, wR_2 = 0.1054
Largest diff. peak/hole/ e Å^{-3}	0.54/-0.42	0.43/-0.23	0.31/-0.23	0.44/-0.35

Table S2: Crystal data and structure refinement for compounds **19** and **20**.

Identification code	**19**	**20**
Empirical formula	$C_{44}H_{79}AlBKN_2O_5Si_2$	$C_{64}H_{112}Al_2B_2N_4O_4Si_4$
Formula weight	849.16	1189.51
Crystal system	monoclinic	orthorhombic
Space group	$P2_1/n$	$Pna2_1$
a/ Å	16.1876(2)	16.1989(3)
b/ Å	18.7705(1)	37.7506(11)
c/ Å	16.5749(1)	11.8985(3)
α/ °	90	90
β/ °	99.514(1)	90
γ/ °	90	90
U/ Å^3	4967.00(7)	7276.1(3)
Z	4	4
ρ_{calc}/ g cm^{-3}	1.136	1.086
μ/ mm^{-1}	1.890	1.327
F(000)	1848.0	2592.0
Crystal size/ mm^3	0.151 × 0.134 × 0.073	0.155 × 0.150 × 0.009
2θ range for data collection/°	7.27 to 145.976	7.79 to 146.048
Index ranges	$-20 \leq h \leq 20$<br>$-23 \leq k \leq 23$<br>$-14 \leq l \leq 20$	$-16 \leq h \leq 19$<br>$-40 \leq k \leq 46$<br>$-11 \leq l \leq 14$
Reflections collected	66966	25592
Independent reflections, R_{int}	9883, 0.0368	10984, 0.0436
Data/restraints/parameters	9883/104/538	10984/1/750
Goodness–of–fit on F^2	1.054	1.038
Final $R1$, $wR2$ [I>=2σ(I)]	R_1 = 0.0467, wR_2 = 0.1311	R_1 = 0.0672, wR_2 = 0.1759
Final $R1$, $wR2$ [all data]	R_1 = 0.0511, wR_2 = 0.1354	R_1 = 0.1037, wR_2 = 0.2009
Largest diff. peak/hole/ e Å^{-3}	0.92/-0.47	0.94/-0.36
Flack parameter	-	0.46(5)

References

1. Dolomanov, O. V.; Bourhis, L.J.; Gildea, R.J.; Howard, J. A. K.; Puschmann, H. *J. Appl. Cryst*. **2009**, *42*, 339-341.
2. Sheldrick, G. M. *Acta Cryst*. **2015**, *A71*, 3-8.
3. Sheldrick, G. M. *Acta Cryst*. **2015**, *C71*, 3-8.
